# Parental attachment and depressive symptoms in pregnancies complicated by twin-twin transfusion syndrome: a cohort study

**DOI:** 10.1186/s12884-019-2679-7

**Published:** 2019-12-31

**Authors:** Fiona L. Mackie, Helen Pattison, Jelena Jankovic, R. Katie Morris, Mark D. Kilby

**Affiliations:** 1 Centre for Women’s & Children’s Health, Institute of Metabolism and Systems Research, University of Birmingham, Birmingham, B15 2TT UK; 2 Birmingham Women’s and Children’s NHS Foundation Trust, Mindelsohn Way, Edgbaston, B15 2TG UK; 3 School of Life and Health Sciences, Aston University, Birmingham, B4 7ET UK; 4 Mother and Baby Unit, Barberry, Birmingham and Solihull Mental Health NHS Foundation Trust, 25 Vincent Drive, Edgbaston, Birmingham, B15 2FG UK

**Keywords:** Attachment, Depression, Maternal, Paternal, Twin pregnancy, TTTS

## Abstract

**Background:**

Twin-twin transfusion syndrome (TTTS) is a highly morbid condition in which treatment exists, but the pregnancy remains high-risk until delivery. It may have serious sequelae, including fetal death, and in the longer term, neurodevelopmental problems. The aim of this study is to assess antenatal and postnatal parental attachment and depressive symptoms in those with pregnancies affected by TTTS.

**Methods:**

Couples attending for fetoscopic laser ablation treatment of TTTS were asked to complete Condon’s Maternal/Paternal Antenatal/Postnatal Attachment Scale as appropriate, and the Edinburgh Depression Scale the day before ablation, 4 weeks post-ablation, and 6–10 weeks postnatally.

**Results:**

25/27 couples completed the pre-ablation questionnaire (median gestational age 19 + 3 weeks [interquartile range 18 + 2–20 + 6]). 8/18 eligible couples returned the post-ablation questionnaire. 5/17 eligible couples returned the postnatal questionnaire. There was no significant difference in parento-fetal attachment when mothers were compared to fathers at each time point, however parento-fetal attachment did increase over time in mothers (*p* = 0.004), but not fathers. Mothers reported more depressive symptoms antenatally compared to fathers (*p* < 0.02), but there was no difference postnatally. 50% women reported Edinburgh Depression Scale scores above the cut-off (≥15) 4 weeks post-ablation. Over time maternal depressive symptoms decreased (*p* = 0.006), however paternal depressive symptoms remained the same.

**Conclusions:**

This is the first attachment and depression study in a UK cohort of parents with pregnancies affected by TTTS. Although this was a small cohort and the questionnaires used had not been validated in these circumstances, the results suggest that centres caring for these couples should be aware of the risk of maternal and paternal antenatal depression, and screen and refer for additional psychological support. Further work is needed in larger cohorts.

**Trial registration:**

ISRCTN 13114861 (retrospectively registered).

## Background

Twin-twin transfusion syndrome (TTTS) is a highly morbid complication of monochorionic (MC) twin pregnancy which occurs due to unbalanced inter-twin blood flow, via placental anastomoses [3]. The gold standard treatment is fetoscopic laser ablation (FLA) whereby the anastomoses are ablated so as to re-balance the inter-twin blood flow [33]. Prognosis is improved dramatically by FLA though approximately 50% will still result in a single intrauterine fetal death (sIUFD), 15% in a double intrauterine fetal death (dIUFD) [26, 35], and in 10–15% neurodevelopmental comorbidity [39]. This unusual scenario where both twins are at risk; and one twin may die and one twin may survive, means that parents can face difficult paradoxical situations. There is a dearth of research on the emotional effects for parents antenatally and postnatally, including on parento-fetal attachment, and depression.

Materno-fetal attachment positively influences maternal health choices antenatally, thus affecting neonatal outcome [1] and also shaping parental postnatal behaviour [10, 34], early infant development [1, 12] and long term child behaviour [12]. Only one study has explored materno-fetal attachment in TTTS pregnancies; this French study found that materno-fetal antenatal attachment increased during pregnancy in mothers with uncomplicated MC and dichorionic twins, but not in mothers with TTTS pregnancies [2].

Antenatally, maternal depression can have fetal and maternal effects [4, 18, 19]. Longer term, maternal antenatal and postnatal depression is negatively associated with child development, and increased behavioural problems [4, 18], as is paternal postnatal depression [30]. In TTTS, Beauquier-Maccotta et al. reported at 20 weeks gestation, when TTTS was diagnosed, the mean Edinburgh postnatal depression scale (EPDS) score of the mothers in the TTTS group was significantly higher than the score of mothers in the gestationally-matched uncomplicated MC twin pregnancy group, with 72% of the TTTS group scoring above the cut-off for major depressive symptoms in French speaking women [2]. At 3 months postnatally the TTTS group reported the highest rate of depression (33%), though sub-group analysis was not performed according to pregnancy outcome. High rates of maternal depressive symptoms antenatally and postnatally when compared to mothers with an uncomplicated MC twin pregnancy have also been reported by a retrospective USA study [15], and at 7 years postnatally in a Belgium study [40], and can effect subsequent pregnancies [28]. Paternal-fetal attachment and depressive symptoms have not been explored in the context of TTTS.

This study investigated maternal and paternal antenatal fetal attachment, postnatal infant attachment and parental depression in pregnancies complicated by TTTS. The authors hypothesise that parento-fetal attachment would be higher in postnatally compared to antenatally, and depressive symptoms would decrease over time.

## Methods

This study received ethical approval from East Midlands Research Ethics Committee (15/EM/0244) and all participants provided informed written consent.

### Participants

Women with monochorionic diamniotic twin pregnancies and their partners, referred to the West Midlands Fetal Medicine Centre (WMFMC) for FLA for TTTS at < 24 weeks gestation were prospectively, consecutively recruited between January 2016 and September 2017; follow-up continued to February 2018. Both the woman and her partner had to attend and provide individual written informed consent to participate. Participants had to be able to read English so as to understand the follow-up postal questionnaires. Women with higher order pregnancies, or whose pregnancies were affected by chromosomal/structural anomalies were not eligible. If a couple suffered a dIUFD or sIUFD prior to FLA, meaning that FLA would not be performed, they were not eligible.

### Measures

#### Attachment

Parental attachment was assessed using four self-reported Attachment Scales [6, 7, 9, 10]:

- Maternal Antenatal Attachment Scale (MAAS)
- Paternal Antenatal Attachment Scale (PAAS)
- Maternal Postnatal Attachment Scale (MPAS)
- Paternal Postnatal Attachment Scale (PPAS)

A higher score denotes greater attachment. Participants were asked to complete the Attachment Scales per pregnancy, not per fetus/infant. In the pre-FLA and post-FLA questionnaires the time point of “2 weeks” was changed to “since the diagnosis of TTTS” (see Additional files 1 and 2).

#### Depressive symptoms

The EPDS was used; a higher score denotes greater depressive symptoms. A cut-off of 15 was used for maternal antenatal depression, and 13 for maternal postnatal depression [25]. A cut-off of 12 was used for paternal antenatal depression [5, 31] and 10 for paternal postnatal depression [24]. The pre-FLA and post-FLA EPDS questionnaires were amended to ask about time “since the diagnosis of TTTS” rather than “the last 7 days”.

#### Mental health history

Questions on current and past mental health problems were asked at each time point (see Additional file 1).

### Procedure

Women and their partners were approached by a trained researcher after they had been consented for FLA by the Fetal Medicine Consultant. This was the day before FLA. The woman and her partner were asked to complete the questionnaire on parento-fetal attachment and depressive symptoms separately. The Attachment and EPDS questionnaires were completed at three time points:

- Pre-FLA: the day prior to FLA (MAAS, PAAS, maternal and paternal EPDS, mental health history)
- Post-FLA: 1 month following FLA (MAAS, PAAS, maternal and paternal EPDS)
- Postnatal: 6–10 weeks following delivery (MPAS, PPAS, maternal and paternal EPDS, mental health history).

Follow-up questionnaires were posted to participants. If follow-up questionnaires were not received, a reminder was sent in the post, and telephone contact attempted. The timing of the questionnaires was related to medical care as 4 weeks post-FLA is when fetal magnetic resonance imaging is advised to assess for brain injury, and 6–10 weeks postnatal allows time for neonatal unit admission if required. If following FLA the couple suffered a dIUFD, double neonatal death, or terminated the whole pregnancy, the post-FLA and/or postnatal questionnaires were not sent out.

It is important to highlight that the time period for the maternal and paternal pre-FLA attachment and EPDS was changed to “since the diagnosis of TTTS” thus the results should be interpreted with caution as the score may be more reflective of an acute adjustment reaction because due to the rapid progression of TTTS “since the diagnosis of TTTS” may equate to 1 day.

### Missing data

See Additional file 2.

### Statistical analysis

See Additional file 2

## Results

### Participant characteristics

Fifty-four women were booked for FLA for TTTS during the 19 month recruitment period: 27 couples were approached and all 27 couples (100%) consented and agreed to participate. One couple was missed in the screening process. The other 26/54 women were ineligible to participate due to: no partner present at appointment (*n* = 16), seen by researcher the morning of FLA therefore insufficient time to complete questionnaire (*n* = 7), dIUFD prior to commencing FLA (n = 1), unable to read English (n = 1), declined to talk to researcher (n = 1). Two couples stated they had been unable to complete the questionnaire prior to FLA and were unable to complete them immediately following FLA, thus the data presented here are for 25 couples (25 mothers, and 25 fathers) (Table 1).

**Table 1:** Participant demographic and pregnancy outcome information

Maternal characteristics (*n* = 25)	
Maternal age median (IQR) years	28.76 (26–32)
Parity n (%)	
Nulliparous	16 (64)
Multiparous	9 (36)
Maternal ethnicity n (%)	
White European	25 (100)
Quintero staging at FLA median (IQR)	3 (2–3)
Gestation at FLA median (IQR) weeks	19 + 3 (18 + 2–20 + 6)
Gestation 1 month following FLA median (IQR) weeks	23 + 5 (22 + 4–25 + 1)
Pregnancy outcome data (*n* = 23 pregnancies that underwent FLA)	
Double survivor n (%)	9 (39.1)
Single survivor n (%)	8 (34.8)
No survivor n (%)	6 (26.1)

23/25 (92%) couples completed all sections of the pre-FLA questionnaire (Additional file 3: Table S1), though 6/25 (24%) of couples completed the pre-FLA questionnaires immediately following FLA. The questionnaire return rate from eligible couples post-FLA was 8/18 (44.4%), and postnatally was 5/17 (29.4%).

### Mental health history of participants

See Additional file 3.

#### Maternal and paternal attachment

**Fig. 1:**
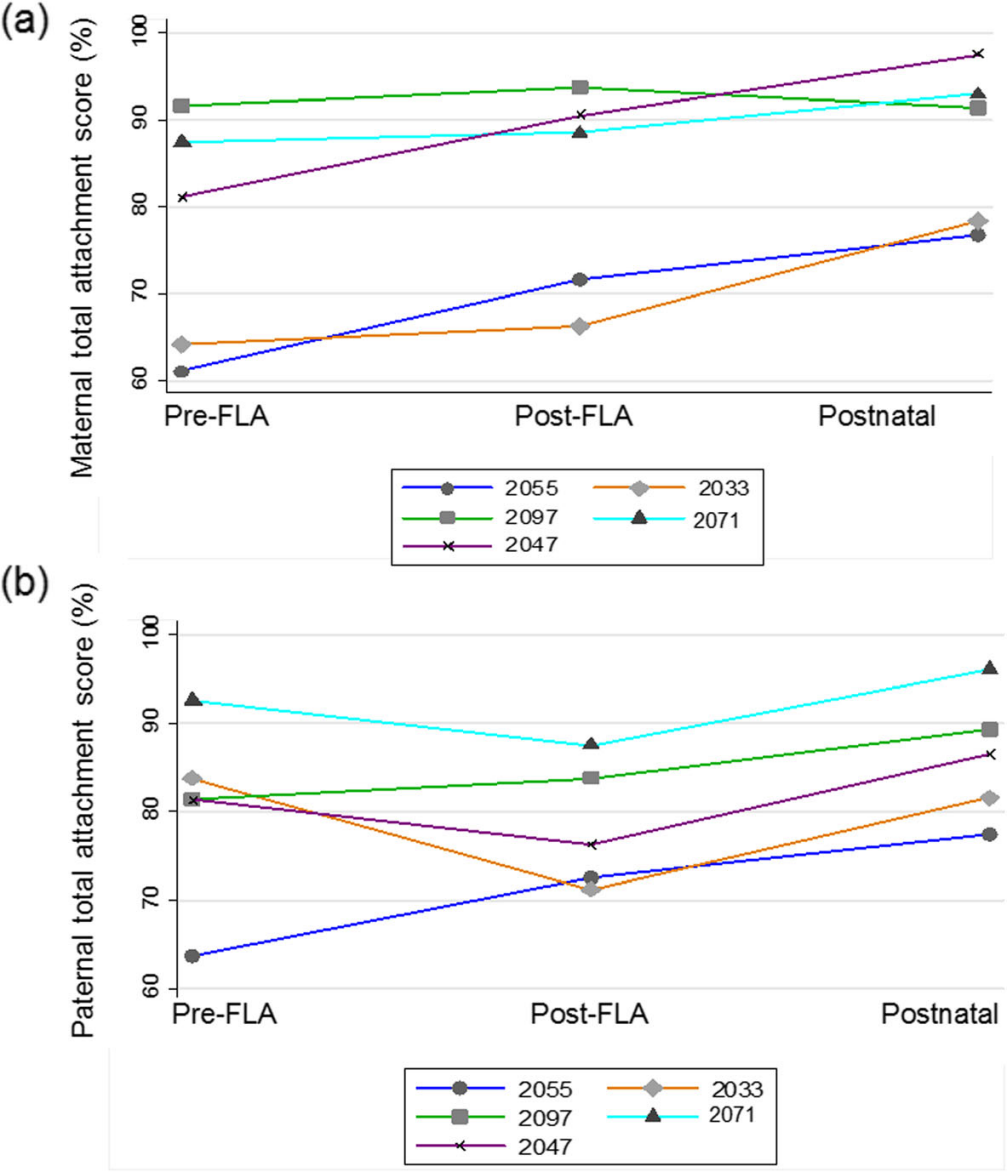
Line plot of individual (**a**) maternal (**b**) paternal Attachment Scale scores as a percentage of the maximum possible score pre-fetoscopic laser ablation (FLA), post-FLA and postnatally(*n* = 5 couples included at all 3 time points) The bottom dark blue line with circle markers is the couple with 1 survivor, the other 4 couples had 2 survivors. *p* < 0.05 maternal attachment pre-FLA to postnatal

There was no significant difference between maternal and paternal attachment scores at each time point (Additional file 3: Table S2, Additional file 3: Figure S1). When maternal and paternal attachment in the 5 couples who completed questionnaires at all 3 time points was examined over time, ANOVA demonstrated a significant difference in maternal attachment, *F* (2, 4) = 7.86, *p* = 0.026 with Greenhouse and Geisser correction of sphericity (ε = 0.736) [17]. Post hoc linear regression revealed a significant increase in maternal attachment from pre-FLA to postnatal (*p* = 0.004) (Fig. 1a). There was no significant change in paternal attachment over time using the Kruskal Wallis test, χ^2^(2) = 2.414, *p* = 0.30 (Fig. 1b). It was not possible to compare couples with 1 survivor to those with 2 survivors due to insufficient numbers.

When the cohort was divided based on existing mental health problems, there were no statistically significant differences between those with and without mental health problems, although the numbers were small (Additional file 3: Table S3). There was no significant difference between those who completed the pre-FLA attachment questionnaire before FLA, and those who completed it immediately after FLA (data not shown).

#### Maternal and paternal depressive symptoms

There was a significant difference between the maternal and paternal EPDS scores pre-FLA and post-FLA, but not postnatally (Table 2, Additional file 3: Figure S2). When the scores were translated into the number of participants above the cut-off for major depressive disorders there was no significant difference between the mothers and fathers at each time point. The time point with the highest proportion of mothers above the cut-off was post-FLA (4/8, 50.0%). Postnatally no mothers had an EPDS score above the cut-off, irrespective of pregnancy outcome but this should be interpreted with caution due to possibly insufficient numbers. The time point with the highest proportion of fathers above the cut-off was pre-FLA (6/23, 26.1%).

**Table 2:** Maternal and paternal Edinburgh Postnatal Depression Scale (EPDS) scores pre-fetoscopic laser ablation (FLA), post-FLA and postnatally

	Maternal pre-FLA (*n* = 24)	Maternal post-FLA (*n* = 8)	Maternal postnatal (n = 5)	Paternal pre-FLA (n = 23)	Paternal post-FLA (n = 7)	Paternal postnatal (n = 5)
Total EPDS score median (IQR)	12.5* (7–17)	12.5† (9.5–17.5)	4 (3–7)	8* (5–11.5)	6† (4.5–9.5)	3 (2–9)
Number of participants above cut-off n/N (%)	10/24 (41.7)	4/8 (50.0)	0/5 (0.0)	6/23 (26.1)	1/7 (14.2)	1/5 (20.0)

*p = 0.01 pre-FLA: maternal vs paternal, †*p* = 0.02 post-FLA: maternal vs paternal

**Fig. 2:**
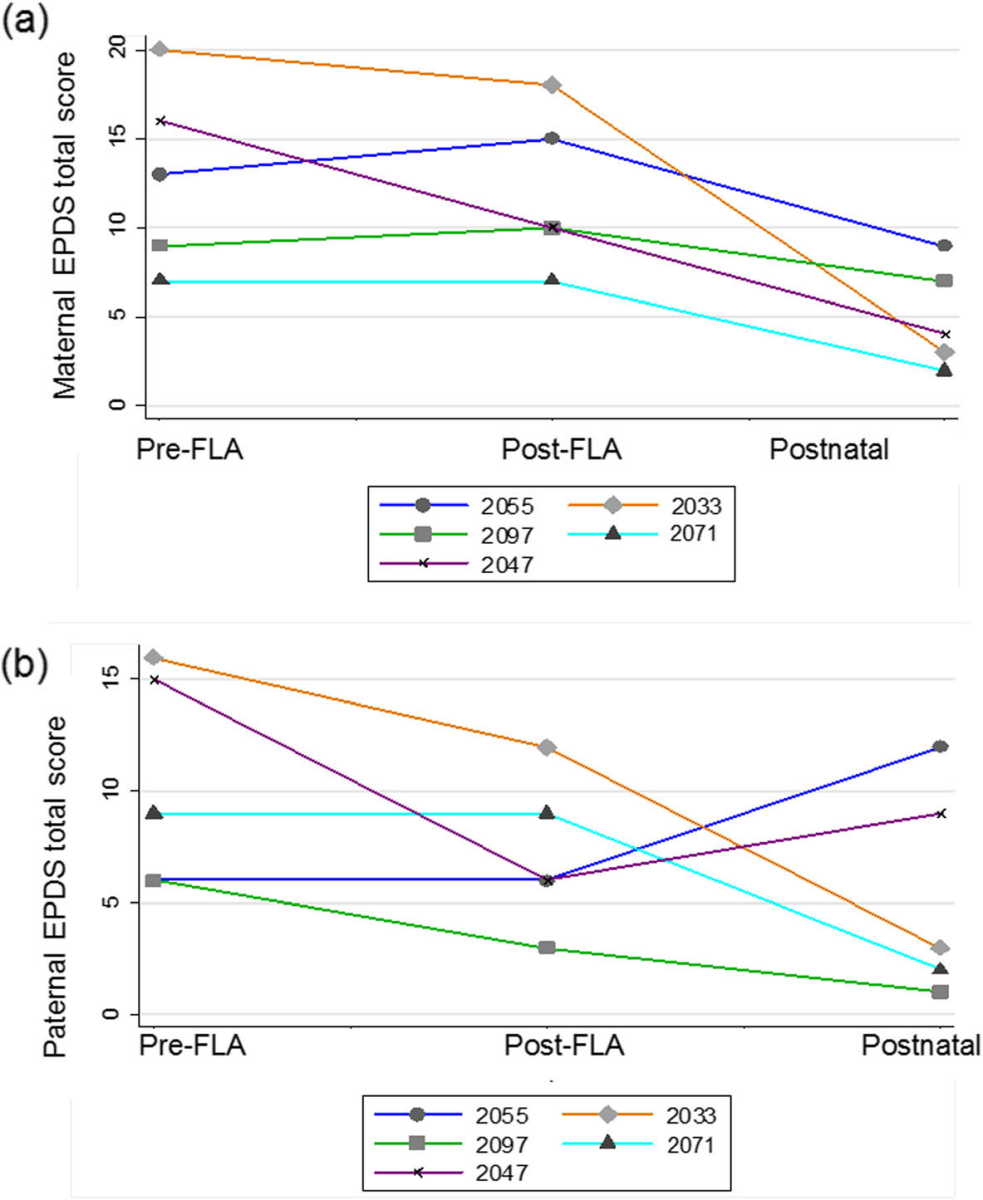
Line plot of individual (**a**) maternal (**b**) paternal Edinburgh Postnatal Depression Scale (EPDS) scores pre-fetoscopic laser ablation (FLA), post-FLA and postnatally(n = 5 couples included at all 3 time points) The bottom dark blue line with circle markers (2055) is the couple with 1 survivor, the other 4 couples had 2 survivors. p < 0.05 maternal EPDS total pre-FLA to postnatal

Postnatally 1/5 (20%) fathers had an EPDS score above the cut-off, which interestingly was the pregnancy with 1 survivor whereas the other 4/5 pregnancies had 2 survivors. When EPDS scores in the 5 couples who completed questionnaires at all 3 time points were examined over time, ANOVA demonstrated a significant difference in maternal depressive symptoms *F* (2, 4) = 8.03, *p* = 0.031 with Greenhouse and Geisser correction of sphericity (ε = 0.655) [17]. Post hoc linear regression revealed a significant decrease in maternal EPDS score from pre-FLA to postnatal (p = 0.006) (Fig. 2a). There was no significant change in paternal depressive symptoms over time using the Kruskal Wallis test, χ^2^(2) = 2.738, *p* = 0.25 (Fig. 2b). It was not possible to formally compare couples with 1 survivor to those with 2 survivors due to insufficient numbers.

Mothers with a history of mental health problems reported significantly greater depressive symptoms post-FLA than mothers with no mental health problems (median EPDS score 23.5 (IQR: 20.75–26.25) vs. 10 (9.25–13.75) respectively) (Additional file 3: Table S4). Fathers with current mental health problems reported significantly greater depressive symptoms pre-FLA than fathers with no history of mental health problems (median EPDS score 12 (IQR: 10–15) vs. 7 (4–9) respectively). However, these increases in EPDS score did not translate into a significant difference in the proportion of mothers and fathers above the cut-offs.

There was a significant difference (*p* = 0.03) in the median maternal EPDS scores between those who completed the pre-FLA questionnaire before the FLA (10.5 [IQR: 7–16.75] 18 mothers) and those who completed it immediately after FLA (19 [IQR: 4.75–22.5] 6 mothers). This did not translate to a difference in the proportion of mothers who scored above the cut-off. There was no difference in the fathers (data not shown).

## Discussion

This is the first UK study to explore attachment and depression in mothers and fathers whose pregnancies have been affected by TTTS. Results in other studies are conflicting regarding whether mothers or fathers report higher levels of attachment in non-TTTS pregnancies. The lack of difference between maternal and paternal attachment may be because the fathers who were more attached were more likely to attend the Fetal Medicine Centre, and agree to participate in the study, or it could be that as fathers have become more involved in family life, paternal attachment levels have increased [37]. Another explanation is that some mothers with high-risk pregnancies employ a protective mechanism and decrease their attachment to the fetuses in the antenatal period [11, 16, 36], consequently mothers are no longer more attached than fathers. This hypothesis is supported by maternal attachment increasing from at time of diagnosis of TTTS to postnatally, but there being no significant increase at the post-FLA time point when the fetuses are still in danger. Beauquier-Maccotta et al. [2] also reported no increase in antenatal maternal attachment in TTTS pregnancies. Interestingly, no change over time was seen in the fathers whereas an increase was seen in the mothers, the latter of which is common in pregnancy [20, 32]. This may reflect that fathers vary in the way they cope with stressful situations, with some fathers employing protective mechanisms and others not.

The decrease in maternal depressive symptoms from diagnosis of TTTS to postnatally was also reported by Beauquier-Maccotta et al. [2] which fits with the pregnancy continuing to be at risk throughout the antenatal period and mothers experiencing relief at the delivery of the survivor(s). Fascinatingly this was not reflected in the fathers with 2/5 EPDS scores increasing postnatally, compared to 0/5 in mothers. This may reveal that fathers experience potentially high-risk situations in a more variable way than mothers. Importantly, when this is combined with no increase in paternal attachment, it highlights the importance of assessing paternal mental health. NHS England has recently recognised that mental health is not routinely evaluated in fathers, where as it is in mothers, and thus they are planning on offering expectant fathers mental health checks [27]. Multiple studies have reported that fathers believe their role is to provide support to the mother and stay in control of the situation; consequently feeling unable to express their emotions [14]. A major strength of this study is the sub-group analysis according to current and post mental health problems. This did demonstrate significantly greater depressive symptoms in mothers and fathers with a history of mental health problems, highlighting the importance of enquiring about mental health problems in mothers and fathers.

Another strength was the use of a validated depression screening tool specific to pregnancy that has been previously used in twin pregnancy. Although the EPDS does not provide a definitive diagnosis of a depressive disorder, it does have a high sensitivity and specificity and is therefore an acceptable screening tool, and is used in routine clinical care. There was a good return of pre-FLA questionnaires, but the proportion of returned post-FLA and postnatal questionnaires was lower than the generally acceptable survey response rate of 60% [22]. Consequently, some of the findings from these time points should be interpreted with caution due to small sample size and the possibility of a type II error. Particularly the finding that no mothers had EPDS scores above the cut-off postnatally, and the sub-group analysis of parents with existing mental health problems, There is also a risk of sampling bias as substantial proportions of the population have not been represented, and selection bias as those very distressed may be less willing to participate, leading to an underestimate of the negative reactions; they may also be less likely to return follow-up questionnaires. The geographical spread of patients may have meant that fewer participants completed the follow-up questionnaires compared to if they had had their antenatal follow-up care at WMFMC. This is an issue with all TTTS studies as FLA needs to be performed by experienced operators, thus treatment is centralised [26].

Although ethnicity was not an exclusion criterion, it was only possible to include one ethnicity in this cohort which is a limitation, but does mean that ethnicity was not a confounding factor [24]. One reason for this may be that Caucasian women have been shown to possibly be at higher risk for TTTS [23]. Continued research in this area, with larger cohorts in different countries using translated questionnaires with appropriate validation is required. Researchers should explore ways of improving the number of post-FLA and postnatal questionnaires returned. One way to capture this information would be to perform interviews with parents, which would also allow information to be obtained regarding the reasons behind the answers to the questions. Given the rarity of TTTS in the general obstetric population, collaboration between Fetal Medicine Centres would be a feasible way to increase sample size, with adequate adjustment for differences in patient populations. Future research should include the exploration of anxiety symptoms, and their relationship with parental attachment and depression, particularly in fathers as anxiety also seems to play a role in parental attachment and depression [8, 13, 21, 38].

This work demonstrates that a high proportion of mothers with TTTS pregnancies score above the EPDS cut-off, particularly 1 month after FLA (50%). Referral centres providing on-going antenatal care for these women should be aware of this risk and screen and refer for additional psychological support as necessary. With regards fathers, health care practitioners should be aware of the variable way fathers experience TTTS, and take the opportunity to inquire about the health of the father. There is a dearth of research on paternal perinatal mental health, and though the EPDS has been validated as a screening tool for fathers, a validated antenatal cut-off for depressive disorders is required.

## Conclusions

In conclusion, this preliminary study of parental antenatal and postnatal attachment and depression in TTTS pregnancies has demonstrated that maternal attachment increases in the postnatal period, and depressive symptoms decrease in the postnatal period, whereas paternal scores do not appear to change over time. The study has also highlighted the importance of health care professionals in referral centres monitoring mothers and fathers following FLA for depressive symptoms, particularly those with a history of mental health problems, and the possible requirement of additional psychological support for high-risk pregnancies undergoing invasive procedures.

## Supplementary information

**Additional file 1.** Questionnaires for parental attachment and depressive symptoms.

**Additional file 2.** Additional methods [29, 41].

**Additional file 3.** Additional results.

## Data Availability

The datasets used and/or analysed during the current study are available from the corresponding author on reasonable request.

## Abbreviations

dIUFD: double intrauterine fetal death
EPDS: Edinburgh postnatal depression scale
FLA: Fetoscopic laser ablation
MAAS: Maternal antenatal attachment scale
MC: Monochorionic
MPAS: Maternal postnatal attachment scale
PAAS: Paternal antenatal attachment scale
PPAS: Paternal postnatal attachment scale
sIUFD: single intrauterine fetal death
TTTS: Twin-twin transfusion syndrome
