# Supplementary material for: Parental attachment and depressive symptoms in pregnancies complicated by twin-twin transfusion syndrome: a cohort study
Source: BMC Pregnancy Childbirth. 2019 Dec 31;20:4. doi: 10.1186/s12884-019-2679-7 (PMC6938629; doi:10.1186/s12884-019-2679-7)
Supplement: Supplementary file 1 — Additional file 1. Questionnaires for parental attachment and depressive symptoms. [file 12884_2019_2679_MOESM1_ESM.docx]

**Additional File 1** Questionnaires for parental attachment and depressive symptoms


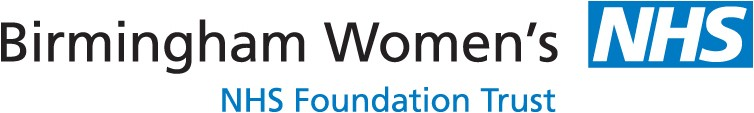


**OMMIT (Optimal Management of Monochorionic Twins) Patient Questionnaire**

**Pre laser Maternal**

**Twin to twin transfusion syndrome is a very nasty complication of pregnancy that if untreated carries a very high risk of mortality for babies. The treatment of fetoscopic laser ablation significantly improves fetal outcomes in many pregnancies, but outcomes may be unpredictable. We realise that having laser surgery is a difficult time for most parents, so we would be very grateful if you could answer the following questions so that we can help other people in the same position as you in the future.**

**We will ask you to complete the questionnaire at different time points (before laser, 1 month after laser, and 6 weeks after you deliver). The aim of our questionnaire is to:**

1. **investigate if undergoing laser surgery affects parental bonding/attachment during pregnancy and after delivery**
2. **learn more about how parents feel who are having laser surgery, so that we are able to provide better support to parents in the future.**

**These questionnaires will be slightly different, but some of the questions are the same so that we can look at how your feelings change during pregnancy, and after you’ve delivered. Please read each question carefully. There are no right or wrong answers. If you would like to discuss any issues raised by this questionnaire with a member of the research team, the contact information is at the bottom.**

**Background Information**

**OMMIT study number __________**

1. **Do you currently have a mental health illness? Yes / No**
   1. **If yes, please specify which condition(s) ________________________________________________________________________________________________________________________________________________**
2. **Do you currently take any medication for a mental health illness? Yes / No**
   1. **If yes, please list any medication ________________________________________________________________________**
3. **Do you currently attend any counselling for a mental health illness? Yes / No**
   1. **If yes, please list any counselling / types of therapy ________________________________________________________________________**
4. **Have you ever had a mental health illness in the past? Yes / No**
   1. **If yes, please specify which condition(s) ________________________________________________________________________________________________________________________________________________**
5. **Did you take any medication for a previous mental health illness? Yes / No**
   1. **If yes, please list any medication ________________________________________________________________________**
6. **Did you attend any counselling for a previous mental health illness? Yes / No**
   1. **If yes, please list any counselling / types of therapy ________________________________________________________________________**

**Condon Fetal Attachment Questions**

**These questions are about your thoughts and feelings about the developing babies. Please tick one box only in answer to each question.**

| **MA1: Since the diagnosis of TTTS I have thought about, or been preoccupied with the babies inside me:** | Please tick |
| --- | --- |
| Almost all the time |  |
| Very frequently |  |
| Frequently |  |
| Occasionally |  |
| Not at all |  |

| **MA2: Since the diagnosis of TTTS when I have spoken about, or thought about the babies inside me I got emotional feelings which were:** | **Please tick** |
| --- | --- |
| Very weak or non-existent |  |
| Fairly weak |  |
| In between strong and weak |  |
| Fairly strong |  |
| Very strong |  |

| **MA3: Since the diagnosis of TTTS my feelings about the babies inside me have been:** | Please tick |
| --- | --- |
| Very positive |  |
| Mainly positive |  |
| Mixed positive and negative |  |
| Mainly negative |  |
| Very negative |  |

| **MA4: Since the diagnosis of TTTS I have the desire to read about or get information about the developing babies. This desire is:** | Please tick |
| --- | --- |
| Very weak or non-existent |  |
| Fairly weak |  |
| Neither strong nor weak |  |
| Moderately strong |  |
| Very strong |  |

| **MA5: Since the diagnosis of TTTS I have been trying to picture in my mind what the developing babies actually look like in my womb:** | Please tick |
| --- | --- |
| Almost all the time |  |
| Very frequently |  |
| Frequently |  |
| Occasionally |  |
| Not at all |  |

| **MA6: Since the diagnosis of TTTS I think of the developing babies mostly as:** | Please tick |
| --- | --- |
| Real little people with special characteristics |  |
| Babies like any other babies |  |
| Human beings |  |
| Living things |  |
| Things not really yet alive |  |

| **MA7: Since the diagnosis of TTTS I have felt that the babies inside me is dependent on me for its well-being:** | Please tick |
| --- | --- |
| Totally |  |
| A great deal |  |
| Moderately |  |
| Slightly |  |
| Not at all |  |

| **MA8: Since the diagnosis of TTTS I have found myself talking to my babies when I am alone:** | Please tick |
| --- | --- |
| Not at all |  |
| Occasionally |  |
| Frequently |  |
| Very frequently |  |
| Almost all the time I am alone |  |

| **MA9: Since the diagnosis of TTTS when I think about (or talk to) my babies inside me, my thoughts:** | Please tick |
| --- | --- |
| Are always tender and loving |  |
| Are mostly tender and loving |  |
| Are a mixture of both tenderness and irritation |  |
| Contain a fair bit of irritation |  |
| Contain a lot of irritation |  |

| **MA10: The picture in my mind of what the babies at this stage actually looks like inside the womb is:** | Please tick |
| --- | --- |
| Very clear |  |
| Fairly clear |  |
| Fairly vague |  |
| Very vague |  |
| I have no idea at all |  |

| **MA11: Since the diagnosis of TTTS when I think about the babies inside me I get feelings which are:** | Please tick |
| --- | --- |
| Very sad |  |
| Moderately sad |  |
| A mixture of happiness and sadness |  |
| Moderately happy |  |
| Very happy |  |

| **MA12: Some pregnant women sometimes get so irritated by the babies inside them that they feel like they want to hurt them or punish them:** | Please tick |
| --- | --- |
| I couldn’t imagine I would ever feel like this |  |
| I could imagine I might sometimes feel like this, but I never actually have |  |
| I have felt like this once or twice myself |  |
| I have occasionally felt like this myself |  |
| I have often felt like this myself |  |
|  | |
| **MA13: Since the diagnosis of TTTS I have felt:** | Please tick |
| Very emotionally distant from my babies |  |
| Moderately emotionally distant from my babies |  |
| Not particularly emotionally close to my babies |  |
| Moderately close emotionally to my babies |  |
| Very close emotionally to my babies |  |

| **MA14: Since the diagnosis of TTTS I have taken care with what I eat to make sure the babies get a good diet:** | Please tick |
| --- | --- |
| Not at all |  |
| Once or twice when I ate |  |
| Occasionally when I ate |  |
| Quite often when I ate |  |
| Every time I ate |  |

| **MA15: When I first see my babies after the birth I expect I will feel:** | Please tick |
| --- | --- |
| Intense affection |  |
| Mostly affection |  |
| Dislike about one or 2 aspects of the babies |  |
| Dislike about quite a few aspects of the baby |  |
| Mostly dislike |  |

| **MA16: When my babies are born I would like to hold the babies:** | Please tick |
| --- | --- |
| Immediately |  |
| After they have been wrapped in a blanket |  |
| After they have been washed |  |
| After a few hours for things to settle down |  |
| The next day |  |

| **MA17: Since the diagnosis of TTTS I have had dreams about the pregnancy or babies:** | Please tick |
| --- | --- |
| Not at all |  |
| Occasionally |  |
| Frequently |  |
| Very frequently |  |
| Almost every night |  |

| **MA18: Since the diagnosis of TTTS I have found myself feeling, or rubbing with my hand, the outside of my stomach where the babies are:** | Please tick |
| --- | --- |
| A lot of times each day |  |
| At least once per day |  |
| Occasionally |  |
| Once only |  |
| Not at all |  |

| **MA19: If the pregnancy was lost at this time (due to miscarriage or other accidental event) without any pain or injury to myself, I expect I would feel:** | Please tick |
| --- | --- |
| Very pleased |  |
| Moderately pleased |  |
| Neutral (i.e. neither sad nor pleased; or mixed feelings) |  |
| Moderately sad |  |
| Very sad |  |

**Edinburgh Postnatal Depression Scale**

**These questions assess your risk for depression. Although it is called the Postnatal Depression Scale, it is also used antenatally before you have your babies. If you score highly, we will inform you and advise you to see your GP for further assessment and support if required.**

**Please tick one box only in answer to each question which comes closest to how you have felt since the diagnosis of TTTS.**

| **EPDSM1: I have been able to laugh and see the funny side of things:** | Please tick |
| --- | --- |
| As much as I always could |  |
| Not quite so much now |  |
| Definitely not so much now |  |
| Not at all |  |

| **EPDSM2: I have looked forward with enjoyment to things:** | Please tick |
| --- | --- |
| As much as I ever did |  |
| Rather less than I used to |  |
| Definitely less than I used to |  |
| Hardly at all |  |

| **EPDSM3: I have blamed myself unnecessarily when things went wrong:** | Please tick |
| --- | --- |
| Yes, most of the time |  |
| Yes, some of the time |  |
| Not very often |  |
| No, never |  |

| **EPDSM4: I have been anxious or worried for no good reason:** | Please tick |
| --- | --- |
| No not at all |  |
| Hardly ever |  |
| Yes, sometimes |  |
| Yes, very often |  |

| **EPDSM5: I have felt scared or panicky for no very good reason:** | Please tick |
| --- | --- |
| Yes, quite a lot |  |
| Not quite so much now |  |
| Definitely not so much now |  |
| Not at all |  |

| **EPDSM6: Things have been getting on top of me:** | Please tick |
| --- | --- |
| Yes, most of the time I haven’t been able to cope at all |  |
| Yes, sometimes I haven’t been coping as well as usual |  |
| No, most of the time I have coped quite well |  |
| No, I have been coping as well as ever |  |

| **EPDSM7: I have been so unhappy that I have had difficulty sleeping:** | Please tick |
| --- | --- |
| Yes, most of the time |  |
| Yes, sometimes |  |
| Not very often |  |
| No, not at all |  |

| **EPDSM8: I have felt sad or miserable:** | Please tick |
| --- | --- |
| Yes, most of the time |  |
| Yes, sometimes |  |
| Not very often |  |
| No, not at all |  |

| **EPDSM9: I have been so unhappy that I have been crying:** | Please tick |
| --- | --- |
| Yes, most of the time |  |
| Yes, quite often |  |
| Only occasionally |  |
| No, never |  |

| **EPDSM10: The thought of harming myself has occurred to me:** | Please tick |
| --- | --- |
| Yes, quite often |  |
| Sometimes |  |
| Hardly ever |  |
| Never |  |

**Thank-you very much for taking the time to complete this questionnaire**

Detail of researchers

1. Dr Fiona Mackie,

Clinical Research Fellow,

Department of Maternal & Fetal Medicine, Floor 3,

Birmingham Women’s Hospital,

Edgbaston, Birmingham, B15 2TG.

Telephone No: 0121-626-4535

1. Professor M.D. Kilby,

Department of Maternal & Fetal Medicine, Floor 3,

Birmingham Women’s Hospital,

Edgbaston, Birmingham, B15 2TG.

Telephone No: 0121-627-2778

Patient Advice Liaison Service

Tel: 0121 627 2747

Email: [pals@bwnft.nhs.uk](mailto:pals@bwnft.nhs.uk) This email address is being protected from spambots. You need JavaScript enabled to view it.

**Fetal Medicine Centre, Birmingham Women’s Foundation Trust,**

Metchley Park Road, Edgbaston, Birmingham. B15 2TG,

Tel.No: 0121 627 2683


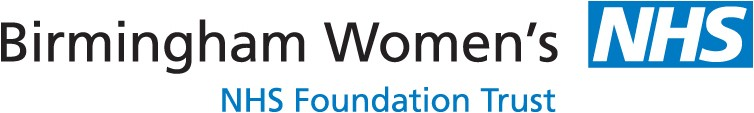


**OMMIT (Optimal Management of Monochorionic Twins) Patient Questionnaire**

**Pre laser Paternal**

**Twin to twin transfusion syndrome is a very nasty complication of pregnancy that if untreated carries a very high risk of mortality for babies. The treatment of fetoscopic laser ablation significantly improves fetal outcomes in many pregnancies, but outcomes may be unpredictable. We realise that having laser surgery is a difficult time for most parents, so we would be very grateful if you could answer the following questions so that we can help other people in the same position as you in the future.**

**We will ask you to complete the questionnaire at different time points (before laser, 1 month after laser, and 6 weeks after your partner delivers). The aim of our questionnaire is to:**

1. **investigate if undergoing laser surgery affects parental bonding/attachment during pregnancy and after delivery**
2. **learn more about how parents feel who are having laser surgery, so that we are able to provide better support to parents in the future.**

**These questionnaires will be slightly different, but some of the questions are the same so that we can look at how your feelings change during pregnancy, and after your partner has delivered. Please read each question carefully. There are no right or wrong answers. If you would like to discuss any issues raised by this questionnaire with a member of the research team, the contact information is at the bottom.**

**Background Information**

**OMMIT study number __________P**

1. **Do you currently have a mental health illness? Yes / No**
   1. **If yes, please specify which condition(s) ________________________________________________________________________________________________________________________________________________**
2. **Do you currently take any medication for a mental health illness? Yes / No**
   1. **If yes, please list any medication ________________________________________________________________________**
3. **Do you currently attend any counselling for a mental health illness? Yes / No**
   1. **If yes, please list any counselling / types of therapy ________________________________________________________________________**
4. **Have you ever had a mental health illness in the past? Yes / No**
   1. **If yes, please specify which condition(s) ________________________________________________________________________________________________________________________________________________**
5. **Did you take any medication for a previous mental health illness? Yes / No**
   1. **If yes, please list any medication ________________________________________________________________________**
6. **Did you attend any counselling for a previous mental health illness? Yes / No**
   1. **If yes, please list any counselling / types of therapy ________________________________________________________________________**

**Condon Fetal Attachment Questions**

**These questions are about your thoughts and feelings about the developing babies. Please tick one box only in answer to each question.**

| **PA1: Since the diagnosis of TTTS I have thought about, or been preoccupied with the babies:** | Please tick |
| --- | --- |
| Almost all the time |  |
| Very frequently |  |
| Frequently |  |
| Occasionally |  |
| Not at all |  |

| **PA2: Since the diagnosis of TTTS when I have spoken about, or thought about the developing babies I got emotional feelings which were:** | Please tick |
| --- | --- |
| Very weak or non-existent |  |
| Fairly weak |  |
| In between strong and weak |  |
| Fairly strong |  |
| Very strong |  |

| **PA3: Since the diagnosis of TTTS my feelings about the developing babies have been:** | Please tick |
| --- | --- |
| Very positive |  |
| Mainly positive |  |
| Mixed positive and negative |  |
| Mainly negative |  |
| Very negative |  |

| **PA4: Since the diagnosis of TTTS I have the desire to read about or get information about the developing babies. This desire is:** | Please tick |
| --- | --- |
| Very weak or non-existent |  |
| Fairly weak |  |
| Neither strong nor weak |  |
| Moderately strong |  |
| Very strong |  |

| **PA5: Since the diagnosis of TTTS I have been trying to picture in my mind what the developing babies actually look like in my partner’s womb:** | Please tick |
| --- | --- |
| Almost all the time |  |
| Very frequently |  |
| Frequently |  |
| Occasionally |  |
| Not at all |  |

| **PA6: Since the diagnosis of TTTS I think of the developing babies mostly as:** | Please tick |
| --- | --- |
| Real little people with special characteristics |  |
| Babies like any other babies |  |
| Human beings |  |
| Living things |  |
| Things not really yet alive |  |

| **PA7: Since the diagnosis of TTTS when I think about the developing babies my thoughts:** | Please tick |
| --- | --- |
| Are always tender and loving |  |
| Are mostly tender and loving |  |
| Are a mixture of both tenderness and irritation |  |
| Contain a fair bit of irritation |  |
| Contain a lot of irritation |  |

| **PA8: Since the diagnosis of TTTS my ideas and possible names for the babies have been:** | Please tick |
| --- | --- |
| Very clear |  |
| Fairly clear |  |
| Fairly vague |  |
| Very vague |  |
| I have no idea at all |  |

| **PA9: Since the diagnosis of TTTS when I think about the developing babies I get feelings which are:** | Please tick |
| --- | --- |
| Very sad |  |
| Moderately sad |  |
| A mixture of happiness and sadness |  |
| Moderately happy |  |
| Very happy |  |

| **PA10: Since the diagnosis of TTTS I have been thinking about what kind of child the baby will grow into:** | Please tick |
| --- | --- |
| Not at all |  |
| Occasionally |  |
| Frequently |  |
| Very frequently |  |
| Almost all the time |  |

| **PA11: Since the diagnosis of TTTS I have felt:** | Please tick |
| --- | --- |
| Very emotionally distant from the babies |  |
| Moderately emotionally distant from the babies |  |
| Not particularly emotionally close to the babies |  |
| Moderately close emotionally to the babies |  |
| Very close emotionally to the babies |  |

| **PA12: When I first see my babies after the birth I expect I will feel:** | Please tick |
| --- | --- |
| Intense affection |  |
| Mostly affection |  |
| Affection, but I expect there may be a few aspects of the babies I will dislike |  |
| I expect there may be quite a few aspects of the babies I will dislike |  |
| I expect I might feel mostly dislike |  |

| **PA13: When the babies are born I would like to hold the babies:** | Please tick |
| --- | --- |
| Immediately |  |
| After they have been wrapped in a blanket |  |
| After they have been washed |  |
| After a few hours for things to settle down |  |
| The next day |  |

| **PA14: Since the diagnosis of TTTS I have had dreams about the pregnancy or babies:** | Please tick |
| --- | --- |
| Not at all |  |
| Occasionally |  |
| Frequently |  |
| Very frequently |  |
| Almost every night |  |

| **PA15: Since the diagnosis of TTTS I have found myself feeling, or rubbing with my hand, the outside of my partner’s stomach where the babies are:** | Please tick |
| --- | --- |
| A lot of times each day |  |
| At least once per day |  |
| Occasionally |  |
| Once only |  |
| Not at all |  |

| **PA16: If the pregnancy was lost at this time (due to miscarriage or other accidental event) without any pain or injury to my partner, I expect I would feel:** | Please tick |
| --- | --- |
| Very pleased |  |
| Moderately pleased |  |
| Neutral (i.e. neither sad nor pleased; or mixed feelings) |  |
| Moderately sad |  |
| Very sad |  |

**Edinburgh Postnatal Depression Scale**

**These questions assess your risk for depression. Although it is called the Postnatal Depression Scale, it is also used antenatally before you have your babies. If you score highly, we will inform you and advise you to see your GP for further assessment and support if required.**

**Please tick one box only in answer to each question which comes closest to how you have felt since the diagnosis of TTTS.**

| **EPDSP1: I have been able to laugh and see the funny side of things:** | Please tick |
| --- | --- |
| As much as I always could |  |
| Not quite so much now |  |
| Definitely not so much now |  |
| Not at all |  |

| **EPDSP2: I have looked forward with enjoyment to things:** | Please tick |
| --- | --- |
| As much as I ever did |  |
| Rather less than I used to |  |
| Definitely less than I used to |  |
| Hardly at all |  |

| **EPDSP3: I have blamed myself unnecessarily when things went wrong:** | Please tick |
| --- | --- |
| Yes, most of the time |  |
| Yes, some of the time |  |
| Not very often |  |
| No, never |  |

| **EPDSP4: I have been anxious or worried for no good reason:** | Please tick |
| --- | --- |
| No not at all |  |
| Hardly ever |  |
| Yes, sometimes |  |
| Yes, very often |  |
|  |  |

| **EPDSP5: I have felt scared or panicky for no very good reason:** | Please tick |
| --- | --- |
| Yes, quite a lot |  |
| Not quite so much now |  |
| Definitely not so much now |  |
| Not at all |  |

| **EPDSP6: Things have been getting on top of me:** | Please tick |
| --- | --- |
| Yes, most of the time I haven’t been able to cope at all |  |
| Yes, sometimes I haven’t been coping as well as usual |  |
| No, most of the time I have coped quite well |  |
| No, I have been coping as well as ever |  |

| **EPDSP7: I have been so unhappy that I have had difficulty sleeping:** | Please tick |
| --- | --- |
| Yes, most of the time |  |
| Yes, sometimes |  |
| Not very often |  |
| No, not at all |  |

| **EPDSP8: I have felt sad or miserable:** | Please tick |
| --- | --- |
| Yes, most of the time |  |
| Yes, sometimes |  |
| Not very often |  |
| No, not at all |  |

| **EPDSP9: I have been so unhappy that I have been crying:** | Please tick |
| --- | --- |
| Yes, most of the time |  |
| Yes, quite often |  |
| Only occasionally |  |
| No, never |  |

| **EPDSP10: The thought of harming myself has occurred to me:** | Please tick |
| --- | --- |
| Yes, quite often |  |
| Sometimes |  |
| Hardly ever |  |
| Never |  |

**Thank-you very much for taking the time to complete this questionnaire**

Detail of researchers

1. Dr Fiona Mackie,

Clinical Research Fellow,

Department of Maternal & Fetal Medicine, Floor 3,

Birmingham Women’s Hospital,

Edgbaston, Birmingham, B15 2TG.

Telephone No: 0121-626-4535

1. Professor M.D. Kilby,

Department of Maternal & Fetal Medicine, Floor 3,

Birmingham Women’s Hospital,

Edgbaston, Birmingham, B15 2TG.

Telephone No: 0121-627-2778

Patient Advice Liaison Service

Tel: 0121 627 2747

Email: [pals@bwnft.nhs.uk](mailto:pals@bwnft.nhs.uk) This email address is being protected from spambots. You need JavaScript enabled to view it.

**Fetal Medicine Centre, Birmingham Women’s Foundation Trust,**

Metchley Park Road, Edgbaston, Birmingham. B15 2TG, Tel.No: 0121 627 2683


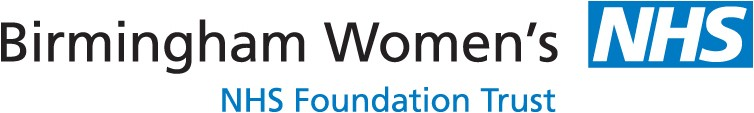


**OMMIT (Optimal Management of Monochorionic Twins) Patient Questionnaire**

**Post laser Maternal**

**We would be grateful if you could complete this questionnaire so that we can help people who need laser surgery in the future.** As you know twin pregnancies are high-risk and people that have twins can have a wide variety of outcomes. Whilst we have tried our best to send you the correct questionnaire depending on your outcome, we may sometimes get this wrong for which we apologise.

**There are no right or wrong answers. If you would like to discuss any issues raised by this questionnaire with a member of the research team, the contact information is at the bottom.**

**Condon Fetal Attachment Questions**

**These questions are about your thoughts and feelings about the developing baby/babies. Please tick one box only in answer to each question.**

| **MA1: Since the diagnosis of TTTS I have thought about, or been preoccupied with the baby/babies inside me:** | Please tick |
| --- | --- |
| Almost all the time |  |
| Very frequently |  |
| Frequently |  |
| Occasionally |  |
| Not at all |  |

| **MA2: Since the diagnosis of TTTS when I have spoken about, or thought about the baby/babies inside me I got emotional feelings which were:** | Please tick |
| --- | --- |
| Very weak or non-existent |  |
| Fairly weak |  |
| In between strong and weak |  |
| Fairly strong |  |
| Very strong |  |

| **MA3: Since the diagnosis of TTTS my feelings about the baby/babies inside me have been:** | Please tick |
| --- | --- |
| Very positive |  |
| Mainly positive |  |
| Mixed positive and negative |  |
| Mainly negative |  |
| Very negative |  |

| **MA4: Since the diagnosis of TTTS I have the desire to read about or get information about the developing baby/babies. This desire is:** | Please tick |
| --- | --- |
| Very weak or non-existent |  |
| Fairly weak |  |
| Neither strong nor weak |  |
| Moderately strong |  |
| Very strong |  |

| **MA5: Since the diagnosis of TTTS I have been trying to picture in my mind what the developing baby/babies actually look like in my womb:** | Please tick |
| --- | --- |
| Almost all the time |  |
| Very frequently |  |
| Frequently |  |
| Occasionally |  |
| Not at all |  |

| **MA6: Since the diagnosis of TTTS I think of the developing baby/babies mostly as:** | Please tick |
| --- | --- |
| Real little people with special characteristics |  |
| Baby/babies like any other baby/babies |  |
| Human beings |  |
| Living things |  |
| Things not really yet alive |  |

| **MA7: Since the diagnosis of TTTS I have felt that the baby/babies inside me is dependent on me for its well-being:** | Please tick |
| --- | --- |
| Totally |  |
| A great deal |  |
| Moderately |  |
| Slightly |  |
| Not at all |  |

| **MA8: Since the diagnosis of TTTS I have found myself talking to my baby/babies when I am alone:** | Please tick |
| --- | --- |
| Not at all |  |
| Occasionally |  |
| Frequently |  |
| Very frequently |  |
| Almost all the time I am alone |  |

| **MA9: Since the diagnosis of TTTS when I think about (or talk to) my baby/babies inside me, my thoughts:** | Please tick |
| --- | --- |
| Are always tender and loving |  |
| Are mostly tender and loving |  |
| Are a mixture of both tenderness and irritation |  |
| Contain a fair bit of irritation |  |
| Contain a lot of irritation |  |

| **MA10: The picture in my mind of what the baby/babies at this stage actually looks like inside the womb is:** | Please tick |
| --- | --- |
| Very clear |  |
| Fairly clear |  |
| Fairly vague |  |
| Very vague |  |
| I have no idea at all |  |
| **MA11: Since the diagnosis of TTTS when I think about the baby/babies inside me I get feelings which are:** | Please tick |
| Very sad |  |
| Moderately sad |  |
| A mixture of happiness and sadness |  |
| Moderately happy |  |
| Very happy |  |

| **MA12: Some pregnant women sometimes get so irritated by the baby/babies inside them that they feel like they want to hurt them or punish them:** | Please tick |
| --- | --- |
| I couldn’t imagine I would ever feel like this |  |
| I could imagine I might sometimes feel like this, but I never actually have |  |
| I have felt like this once or twice myself |  |
| I have occasionally felt like this myself |  |
| I have often felt like this myself |  |
|  | |
| **MA13: Since the diagnosis of TTTS I have felt:** | Please tick |
| Very emotionally distant from my baby/babies |  |
| Moderately emotionally distant from my baby/babies |  |
| Not particularly emotionally close to my baby/babies |  |
| Moderately close emotionally to my baby/babies |  |
| Very close emotionally to my baby/babies |  |

| **MA14: Since the diagnosis of TTTS I have taken care with what I eat to make sure the baby/babies get a good diet:** | Please tick |
| --- | --- |
| Not at all |  |
| Once or twice when I ate |  |
| Occasionally when I ate |  |
| Quite often when I ate |  |
| Every time I ate |  |

| **MA15: When I first see my baby/babies after the birth I expect I will feel:** | Please tick |
| --- | --- |
| Intense affection |  |
| Mostly affection |  |
| Dislike about one or 2 aspects of the baby/babies |  |
| Dislike about quite a few aspects of the baby |  |
| Mostly dislike |  |

| **MA16: When my baby/babies are born I would like to hold the baby/babies:** | Please tick |
| --- | --- |
| Immediately |  |
| After they have been wrapped in a blanket |  |
| After they have been washed |  |
| After a few hours for things to settle down |  |
| The next day |  |

| **MA17: Since the diagnosis of TTTS I have had dreams about the pregnancy or baby/babies:** | Please tick |
| --- | --- |
| Not at all |  |
| Occasionally |  |
| Frequently |  |
| Very frequently |  |
| Almost every night |  |

| **MA18: Since the diagnosis of TTTS I have found myself feeling, or rubbing with my hand, the outside of my stomach where the baby/babies are:** | Please tick |
| --- | --- |
| A lot of times each day |  |
| At least once per day |  |
| Occasionally |  |
| Once only |  |
| Not at all |  |

| **MA19: If the pregnancy was lost at this time (due to miscarriage or other accidental event) without any pain or injury to myself, I expect I would feel:** | Please tick |
| --- | --- |
| Very pleased |  |
| Moderately pleased |  |
| Neutral (i.e. neither sad nor pleased; or mixed feelings) |  |
| Moderately sad |  |
| Very sad |  |

**Edinburgh Postnatal Depression Scale**

**These questions assess your risk for depression. Although it is called the Postnatal Depression Scale, it is also used antenatally before you have your baby/babies. If you score highly, we will inform you and advise you to see your GP for further assessment and support if required.**

**Please tick one box only in answer to each question which comes closest to how you have felt since the diagnosis of TTTS.**

| **EPDSM1: I have been able to laugh and see the funny side of things:** | Please tick |
| --- | --- |
| As much as I always could |  |
| Not quite so much now |  |
| Definitely not so much now |  |
| Not at all |  |

| **EPDSM2: I have looked forward with enjoyment to things:** | Please tick |
| --- | --- |
| As much as I ever did |  |
| Rather less than I used to |  |
| Definitely less than I used to |  |
| Hardly at all |  |

| **EPDSM3: I have blamed myself unnecessarily when things went wrong:** | Please tick |
| --- | --- |
| Yes, most of the time |  |
| Yes, some of the time |  |
| Not very often |  |
| No, never |  |

| **EPDSM4: I have been anxious or worried for no good reason:** | Please tick |
| --- | --- |
| No not at all |  |
| Hardly ever |  |
| Yes, sometimes |  |
| Yes, very often |  |

| **EPDSM5: I have felt scared or panicky for no very good reason:** | Please tick |
| --- | --- |
| Yes, quite a lot |  |
| Not quite so much now |  |
| Definitely not so much now |  |
| Not at all |  |

| **EPDSM6: Things have been getting on top of me:** | Please tick |
| --- | --- |
| Yes, most of the time I haven’t been able to cope at all |  |
| Yes, sometimes I haven’t been coping as well as usual |  |
| No, most of the time I have coped quite well |  |
| No, I have been coping as well as ever |  |

| **EPDSM7: I have been so unhappy that I have had difficulty sleeping:** | Please tick |
| --- | --- |
| Yes, most of the time |  |
| Yes, sometimes |  |
| Not very often |  |
| No, not at all |  |

| **EPDSM8: I have felt sad or miserable:** | Please tick |
| --- | --- |
| Yes, most of the time |  |
| Yes, sometimes |  |
| Not very often |  |
| No, not at all |  |

| **EPDSM9: I have been so unhappy that I have been crying:** | Please tick |
| --- | --- |
| Yes, most of the time |  |
| Yes, quite often |  |
| Only occasionally |  |
| No, never |  |

| **EPDSM10: The thought of harming myself has occurred to me:** | Please tick |
| --- | --- |
| Yes, quite often |  |
| Sometimes |  |
| Hardly ever |  |
| Never |  |

**Thank-you very much for taking the time to complete this questionnaire**

Detail of researchers

1. Dr Fiona Mackie,

Clinical Research Fellow,

Department of Maternal & Fetal Medicine, Floor 3,

Birmingham Women’s Hospital,

Edgbaston, Birmingham, B15 2TG.

Telephone No: 0121-626-4535

1. Professor M.D. Kilby,

Department of Maternal & Fetal Medicine, Floor 3,

Birmingham Women’s Hospital,

Edgbaston, Birmingham, B15 2TG.

Telephone No: 0121-627-2778

Patient Advice Liaison Service

Tel: 0121 627 2747

Email: [pals@bwnft.nhs.uk](mailto:pals@bwnft.nhs.uk) This email address is being protected from spambots. You need JavaScript enabled to view it.

**Fetal Medicine Centre, Birmingham Women’s Foundation Trust,**

Metchley Park Road, Edgbaston, Birmingham. B15 2TG, Tel.No: 0121 627 2683


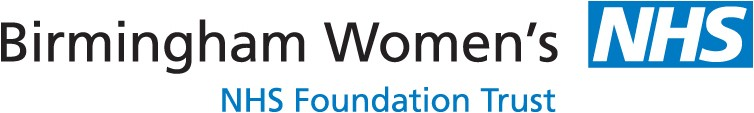


**OMMIT (Optimal Management of Monochorionic Twins) Patient Questionnaire**

**Post laser Paternal**

**We would be grateful if you could complete this questionnaire so that we can help people who need laser surgery in the future.** As you know twin pregnancies are high-risk and people that have twins can have a wide variety of outcomes. Whilst we have tried our best to send you the correct questionnaire depending on your outcome, we may sometimes get this wrong for which we apologise.

**There are no right or wrong answers. If you would like to discuss any issues raised by this questionnaire with a member of the research team, the contact information is at the bottom.**

**Condon Fetal Attachment Questions**

**These questions are about your thoughts and feelings about the developing baby/babies. Please tick one box only in answer to each question.**

| **PA1: Since the diagnosis of TTTS I have thought about, or been preoccupied with the baby/babies:** | Please tick |
| --- | --- |
| Almost all the time |  |
| Very frequently |  |
| Frequently |  |
| Occasionally |  |
| Not at all |  |

| **PA2: Since the diagnosis of TTTS when I have spoken about, or thought about the developing baby/babies I got emotional feelings which were:** | Please tick |
| --- | --- |
| Very weak or non-existent |  |
| Fairly weak |  |
| In between strong and weak |  |
| Fairly strong |  |
| Very strong |  |
|  |  |
| **PA3: Since the diagnosis of TTTS my feelings about the developing baby/babies have been:** | Please tick |
| Very positive |  |
| Mainly positive |  |
| Mixed positive and negative |  |
| Mainly negative |  |
| Very negative |  |

| **PA4: Since the diagnosis of TTTS I have the desire to read about or get information about the developing baby/babies. This desire is:** | Please tick |
| --- | --- |
| Very weak or non-existent |  |
| Fairly weak |  |
| Neither strong nor weak |  |
| Moderately strong |  |
| Very strong |  |

| **PA5: Since the diagnosis of TTTS I have been trying to picture in my mind what the developing baby/babies actually look like in my partner’s womb:** | Please tick |
| --- | --- |
| Almost all the time |  |
| Very frequently |  |
| Frequently |  |
| Occasionally |  |
| Not at all |  |

| **PA6: Since the diagnosis of TTTS I think of the developing baby/babies mostly as:** | Please tick |
| --- | --- |
| Real little people with special characteristics |  |
| Baby/babies like any other baby/babies |  |
| Human beings |  |
| Living things |  |
| Things not really yet alive |  |

| **PA7: Since the diagnosis of TTTS when I think about the developing baby/babies my thoughts:** | Please tick |
| --- | --- |
| Are always tender and loving |  |
| Are mostly tender and loving |  |
| Are a mixture of both tenderness and irritation |  |
| Contain a fair bit of irritation |  |
| Contain a lot of irritation |  |

| **PA8: Since the diagnosis of TTTS my ideas and possible names for the baby/babies have been:** | Please tick |
| --- | --- |
| Very clear |  |
| Fairly clear |  |
| Fairly vague |  |
| Very vague |  |
| I have no idea at all |  |

| **PA9: Since the diagnosis of TTTS when I think about the developing baby/babies I get feelings which are:** | Please tick |
| --- | --- |
| Very sad |  |
| Moderately sad |  |
| A mixture of happiness and sadness |  |
| Moderately happy |  |
| Very happy |  |

| **PA10: Since the diagnosis of TTTS I have been thinking about what kind of child the baby will grow into:** | Please tick |
| --- | --- |
| Not at all |  |
| Occasionally |  |
| Frequently |  |
| Very frequently |  |
| Almost all the time |  |
|  | |
| **PA11: Since the diagnosis of TTTS I have felt:** | Please tick |
| Very emotionally distant from the baby/babies |  |
| Moderately emotionally distant from the baby/babies |  |
| Not particularly emotionally close to the baby/babies |  |
| Moderately close emotionally to the baby/babies |  |
| Very close emotionally to the baby/babies |  |

| **PA12: When I first see my baby/babies after the birth I expect I will feel:** | Please tick |
| --- | --- |
| Intense affection |  |
| Mostly affection |  |
| Affection, but I expect there may be a few aspects of the baby/babies I will dislike |  |
| I expect there may be quite a few aspects of the baby/babies I will dislike |  |
| I expect I might feel mostly dislike |  |

| **PA13: When the baby/babies are born I would like to hold the baby/babies:** | Please tick |
| --- | --- |
| Immediately |  |
| After they have been wrapped in a blanket |  |
| After they have been washed |  |
| After a few hours for things to settle down |  |
| The next day |  |

| **PA14: Since the diagnosis of TTTS I have had dreams about the pregnancy or baby/babies:** | Please tick |
| --- | --- |
| Not at all |  |
| Occasionally |  |
| Frequently |  |
| Very frequently |  |
| Almost every night |  |

| **PA15: Since the diagnosis of TTTS I have found myself feeling, or rubbing with my hand, the outside of my partner’s stomach where the baby/babies are:** | Please tick |
| --- | --- |
| A lot of times each day |  |
| At least once per day |  |
| Occasionally |  |
| Once only |  |
| Not at all |  |

| **PA16: If the pregnancy was lost at this time (due to miscarriage or other accidental event) without any pain or injury to my partner, I expect I would feel:** | Please tick |
| --- | --- |
| Very pleased |  |
| Moderately pleased |  |
| Neutral (i.e. neither sad nor pleased; or mixed feelings) |  |
| Moderately sad |  |
| Very sad |  |

**Edinburgh Postnatal Depression Scale**

**These questions assess your risk for depression. Although it is called the Postnatal Depression Scale, it is also used antenatally before you have your baby/babies. If you score highly, we will inform you and advise you to see your GP for further assessment and support if required.**

**Please tick one box only in answer to each question which comes closest to how you have felt since the diagnosis of TTTS.**

| **EPDSP1: I have been able to laugh and see the funny side of things:** | Please tick |
| --- | --- |
| As much as I always could |  |
| Not quite so much now |  |
| Definitely not so much now |  |
| Not at all |  |

| **EPDSP2: I have looked forward with enjoyment to things:** | Please tick |
| --- | --- |
| As much as I ever did |  |
| Rather less than I used to |  |
| Definitely less than I used to |  |
| Hardly at all |  |

| **EPDSP3: I have blamed myself unnecessarily when things went wrong:** | Please tick |
| --- | --- |
| Yes, most of the time |  |
| Yes, some of the time |  |
| Not very often |  |
| No, never |  |

| **EPDSP4: I have been anxious or worried for no good reason:** | Please tick |
| --- | --- |
| No not at all |  |
| Hardly ever |  |
| Yes, sometimes |  |
| Yes, very often |  |

| **EPDSP5: I have felt scared or panicky for no very good reason:** | Please tick |
| --- | --- |
| Yes, quite a lot |  |
| Not quite so much now |  |
| Definitely not so much now |  |
| Not at all |  |

| **EPDSP6: Things have been getting on top of me:** | Please tick |
| --- | --- |
| Yes, most of the time I haven’t been able to cope at all |  |
| Yes, sometimes I haven’t been coping as well as usual |  |
| No, most of the time I have coped quite well |  |
| No, I have been coping as well as ever |  |

| **EPDSP7: I have been so unhappy that I have had difficulty sleeping:** | Please tick |
| --- | --- |
| Yes, most of the time |  |
| Yes, sometimes |  |
| Not very often |  |
| No, not at all |  |

| **EPDSP8: I have felt sad or miserable:** | Please tick |
| --- | --- |
| Yes, most of the time |  |
| Yes, sometimes |  |
| Not very often |  |
| No, not at all |  |

| **EPDSP9: I have been so unhappy that I have been crying:** | Please tick |
| --- | --- |
| Yes, most of the time |  |
| Yes, quite often |  |
| Only occasionally |  |
| No, never |  |

| **EPDSP10: The thought of harming myself has occurred to me:** | Please tick |
| --- | --- |
| Yes, quite often |  |
| Sometimes |  |
| Hardly ever |  |
| Never |  |

**Thank-you very much for taking the time to complete this questionnaire**

Detail of researchers

1. Dr Fiona Mackie,

Clinical Research Fellow,

Department of Maternal & Fetal Medicine, Floor 3,

Birmingham Women’s Hospital,

Edgbaston, Birmingham, B15 2TG.

Telephone No: 0121-626-4535

1. Professor M.D. Kilby,

Department of Maternal & Fetal Medicine, Floor 3,

Birmingham Women’s Hospital,

Edgbaston, Birmingham, B15 2TG.

Telephone No: 0121-627-2778

Patient Advice Liaison Service

Tel: 0121 627 2747

Email: [pals@bwnft.nhs.uk](mailto:pals@bwnft.nhs.uk) This email address is being protected from spambots. You need JavaScript enabled to view it.

**Fetal Medicine Centre, Birmingham Women’s Foundation Trust,**

Metchley Park Road, Edgbaston, Birmingham. B15 2TG, Tel.No: 0121 627 2683


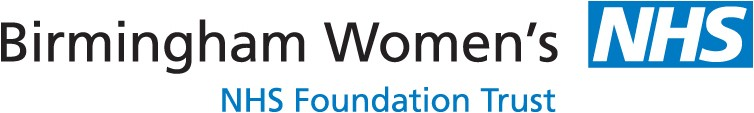


**OMMIT (Optimal Management of Monochorionic Twins) Patient Questionnaire**

**Postnatal Maternal**

As you know twin pregnancies are high-risk and people that have twins can have a wide variety of outcomes. Whilst we have tried our best to send you the correct questionnaire depending on your outcome, we may sometimes get this wrong for which we apologise. Thank-you very much for being part of our study, which we hope will improve care for people in your position in the future.

**Please read each question carefully. There are no right or wrong answers. If you would like to discuss any issues raised by this questionnaire with a member of the research team, the contact information is at the bottom.**

**Background Information**

**OMMIT study number __________P**

1. **Since the first time we asked you this question before your laser surgery, have you been diagnosed with a mental health illness? Yes / No**
   1. **If yes, please specify which condition(s) ________________________________________________________________________________________________________________________________________________**
2. **Do you currently take any medication for a mental health illness? Yes / No**
   1. **If yes, please list any medication ________________________________________________________________________**
3. **Do you currently attend any counselling for a mental health illness? Yes / No**
   1. **If yes, please list any counselling / types of therapy ________________________________________________________________________**

**Condon Fetal Attachment Questions**

**These questions are about your thoughts and feelings about your baby/babies. Please tick one box only in answer to each question.**

| **MP1: When I am caring for the baby/babies, I get feelings of annoyance or irritation:** | Please tick |
| --- | --- |
| Very frequently |  |
| Frequently |  |
| Occasionally |  |
| Very rarely |  |
| Never |  |

| **MP2: When I am caring for the baby/babies I get feelings that they are deliberately being difficult or trying to upset me:** | Please tick |
| --- | --- |
| Very frequently |  |
| Frequently |  |
| Occasionally |  |
| Very rarely |  |
| Never |  |

| **MP3: Over the last 2 weeks I would describe my feelings for the baby/babies as:** | Please tick |
| --- | --- |
| Dislike |  |
| No strong feelings towards the baby/babies |  |
| Slight affection |  |
| Moderate affection |  |
| Intense affection |  |

| **MP4: Regarding my overall level of interaction with the baby/babies I:** | Please tick |
| --- | --- |
| Feel very guilty that I am not more involved |  |
| Feel moderately guilty that I am not more involved |  |
| Feel slightly guilty that I am not more involved |  |
| I don’t have any guilty feelings regarding this |  |

| **MP5: When I interact with the baby/babies I feel:** | Please tick |
| --- | --- |
| Very incompetent and lacking in confidence |  |
| Moderately incompetent and lacking in confidence |  |
| Moderately competent and confident |  |
| Very competent and confident |  |

| **MP6: When I am with the baby/babies I feel tense and anxious:** | Please tick |
| --- | --- |
| Very frequently |  |
| Frequently |  |
| Occasionally |  |
| Almost never |  |

| **MP7: When I am with the baby/babies and other people are present, I feel proud of the baby/babies:** | Please tick |
| --- | --- |
| Very frequently |  |
| Frequently |  |
| Occasionally |  |
| Almost never |  |

| **MP8: I try to involve myself as much as I possibly can PLAYING with the baby/babies:** | Please tick |
| --- | --- |
| This is true |  |
| This is untrue |  |

| **MP9: When I have to leave the baby/babies:** | Please tick |
| --- | --- |
| I usually feel rather sad (or it’s difficult to leave) |  |
| I often feel rather sad (or it’s difficult to leave) |  |
| I have mixed feelings of both sadness and relief |  |
| I often feel rather relieved (and it’s easy to leave) |  |
| I usually feel rather relieved (and it’s easy to leave) |  |

| **MP10: When I am with the baby/babies:** | Please tick |
| --- | --- |
| I always get a lot of enjoyment/satisfaction |  |
| I frequently get a lot of enjoyment/satisfaction |  |
| I occasionally get a lot of enjoyment/satisfaction |  |
| I very rarely get a lot of enjoyment/satisfaction |  |

| **MP11: When I am not with the baby/babies, I find myself thinking about the baby/babies:** | Please tick |
| --- | --- |
| Almost all the time |  |
| Very frequently |  |
| Frequently |  |
| Occasionally |  |
| Not at all |  |

| **MP12: When I am with the baby/babies:** | Please tick |
| --- | --- |
| I usually try to prolong the time I spend with him/her/them |  |
| I usually try to shorten the time I spend with him/her/them |  |

| **MP13: When I have been away from the baby/babies for a while and I am about to be with him/her/them again, I usually feel:** | Please tick |
| --- | --- |
| Intense pleasure at the idea |  |
| Moderate pleasure at the idea |  |
| Mild pleasure at the idea |  |
| No feelings at all about the idea |  |
| Negative feelings about the idea |  |

| **MP14: I now think of the baby/babies as:** | Please tick |
| --- | --- |
| Very much as my own baby/babies |  |
| A bit like my own baby/babies |  |
| Not yet really my own baby/babies |  |

| **MP15: Regarding the things that we have had to give up because of the baby/babies:** | Please tick |
| --- | --- |
| I find that I resent it quite a lot |  |
| I find that I resent it a moderate amount |  |
| I find that I resent it a bit |  |
| I don’t resent it at all |  |

| **MP16: Over the past three months, I have felt that I do not have enough time for myself or to pursue my own interests:** | Please tick |
| --- | --- |
| Almost all the time |  |
| Very frequently |  |
| Occasionally |  |
| Not at all |  |

| **MP17: Taking care of these baby/babies is a heavy burden of responsibility. I believe this is:** | Please tick |
| --- | --- |
| Very much so |  |
| Somewhat so |  |
| Slightly so |  |
| Not at all |  |

| **MP18: I trust my own judgement in deciding what the baby/babies needs:** | Please tick |
| --- | --- |
| Almost never |  |
| Occasionally |  |
| Most of the time |  |
| Almost all the time |  |

| **MP19: Usually when I am with the baby/babies:** | Please tick |
| --- | --- |
| I am very impatient |  |
| I am a bit impatient |  |
| I am moderately patient |  |
| I am extremely patient |  |

**Edinburgh Postnatal Depression Scale**

**These questions assess your risk for depression. If you score highly, we will inform you and advise you to see your GP for further assessment and support if required.**

**Please tick one box only in answer to each question which comes closest to how you have felt in the last 7 days.**

| **EPDSM1: I have been able to laugh and see the funny side of things:** | Please tick |
| --- | --- |
| As much as I always could |  |
| Not quite so much now |  |
| Definitely not so much now |  |
| Not at all |  |

| **EPDSM2: I have looked forward with enjoyment to things:** | Please tick |
| --- | --- |
| As much as I ever did |  |
| Rather less than I used to |  |
| Definitely less than I used to |  |
| Hardly at all |  |

| **EPDSM3: I have blamed myself unnecessarily when things went wrong:** | Please tick |
| --- | --- |
| Yes, most of the time |  |
| Yes, some of the time |  |
| Not very often |  |
| No, never |  |

| **EPDSM4: I have been anxious or worried for no good reason:** | Please tick |
| --- | --- |
| No not at all |  |
| Hardly ever |  |
| Yes, sometimes |  |
| Yes, very often |  |

| **EPDSM5: I have felt scared or panicky for no very good reason:** | Please tick |
| --- | --- |
| Yes, quite a lot |  |
| Not quite so much now |  |
| Definitely not so much now |  |
| Not at all |  |

| **EPDSM6: Things have been getting on top of me:** | Please tick |
| --- | --- |
| Yes, most of the time I haven’t been able to cope at all |  |
| Yes, sometimes I haven’t been coping as well as usual |  |
| No, most of the time I have coped quite well |  |
| No, I have been coping as well as ever |  |

| **EPDSM7: I have been so unhappy that I have had difficulty sleeping:** | Please tick |
| --- | --- |
| Yes, most of the time |  |
| Yes, sometimes |  |
| Not very often |  |
| No, not at all |  |

| **EPDSM8: I have felt sad or miserable:** | Please tick |
| --- | --- |
| Yes, most of the time |  |
| Yes, sometimes |  |
| Not very often |  |
| No, not at all |  |

| **EPDSM9: I have been so unhappy that I have been crying:** | Please tick |
| --- | --- |
| Yes, most of the time |  |
| Yes, quite often |  |
| Only occasionally |  |
| No, never |  |

| **EPDSM10: The thought of harming myself has occurred to me:** | Please tick |
| --- | --- |
| Yes, quite often |  |
| Sometimes |  |
| Hardly ever |  |
| Never |  |

**Thank-you very much for taking the time to complete this questionnaire and being part of this study**

Detail of researchers

1. Dr Fiona Mackie,

Clinical Research Fellow,

Department of Maternal & Fetal Medicine, Floor 3,

Birmingham Women’s Hospital,

Edgbaston, Birmingham, B15 2TG.

Telephone No: 0121-626-4535

1. Professor M.D. Kilby,

Department of Maternal & Fetal Medicine, Floor 3,

Birmingham Women’s Hospital,

Edgbaston, Birmingham, B15 2TG.

Telephone No: 0121-627-2778

Patient Advice Liaison Service

Tel: 0121 627 2747

Email: [pals@bwnft.nhs.uk](mailto:pals@bwnft.nhs.uk) This email address is being protected from spambots. You need JavaScript enabled to view it.

**Fetal Medicine Centre, Birmingham Women’s Foundation Trust,**

Metchley Park Road, Edgbaston, Birmingham. B15 2TG, Tel.No: 0121 627 2683


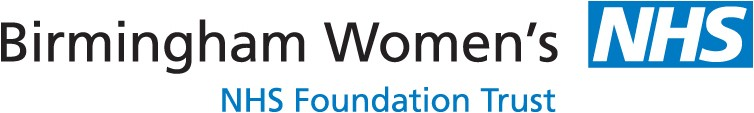


**OMMIT (Optimal Management of Monochorionic Twins) Patient Questionnaire**

**Postnatal Paternal**

As you know twin pregnancies are high-risk and people that have twins can have a wide variety of outcomes. Whilst we have tried our best to send you the correct questionnaire depending on your outcome, we may sometimes get this wrong for which we apologise. Thank-you very much for being part of our study, which we hope will improve care for people in your position in the future.

**Please read each question carefully. There are no right or wrong answers. If you would like to discuss any issues raised by this questionnaire with a member of the research team, the contact information is at the bottom.**

**Background Information**

**OMMIT study number __________P**

1. **Since the first time we asked you this question before laser surgery, have you been diagnosed with a mental health illness? Yes / No**
   1. **If yes, please specify which condition(s) ________________________________________________________________________________________________________________________________________________**
2. **Do you currently take any medication for a mental health illness? Yes / No**
   1. **If yes, please list any medication ________________________________________________________________________**
3. **Do you currently attend any counselling for a mental health illness? Yes / No**
   1. **If yes, please list any counselling / types of therapy ________________________________________________________________________**

**Condon Fetal Attachment Questions**

**These questions are about your thoughts and feelings about your baby/babies. Please tick one box only in answer to each question.**

| **PP1: When I am caring for the baby/babies, I get feelings of annoyance or irritation:** | Please tick |
| --- | --- |
| Very frequently |  |
| Frequently |  |
| Occasionally |  |
| Very rarely |  |
| Never |  |

| **PP2: When I am caring for the baby/babies I get feelings that they are deliberately being difficult or trying to upset me:** | Please tick |
| --- | --- |
| Very frequently |  |
| Frequently |  |
| Occasionally |  |
| Very rarely |  |
| Never |  |

| **PP3: Over the last 2 weeks I would describe my feelings for the baby/babies as:** | Please tick |
| --- | --- |
| Dislike |  |
| No strong feelings towards the baby/babies |  |
| Slight affection |  |
| Moderate affection |  |
| Intense affection |  |

| **PP4: I can understand what my baby/babies needs or wants:** | Please tick |
| --- | --- |
| Almost always |  |
| Usually |  |
| Sometimes |  |
| Rarely |  |
| Almost never |  |

| **PP5: Regarding my overall level of interaction with the baby/babies I believe I am:** | Please tick |
| --- | --- |
| Much more involved than most fathers in my position |  |
| Somewhat more involved than most fathers in my position |  |
| Involved to the same extent as most fathers in my position |  |
| Somewhat less involved than most fathers in my position |  |
| Much less involved than most fathers in my position |  |

| **PP6: When I am with the baby/babies I feel bored:** | Please tick |
| --- | --- |
| Very frequently |  |
| Frequently |  |
| Occasionally |  |
| Almost never |  |

| **PP7: When I am with the baby/babies and other people are present, I feel proud of the baby/babies:** | Please tick |
| --- | --- |
| Very frequently |  |
| Frequently |  |
| Occasionally |  |
| Almost never |  |

| **PP8: I try to involve myself as much as possible in child care and looking after the baby/babies:** | Please tick |
| --- | --- |
| This is true |  |
| This is untrue |  |

| **PP9: I find myself talking to people (other than my partner) about the baby/babies:** | Please tick |
| --- | --- |
| Many times each day |  |
| A few times each day |  |
| Once or twice a day |  |
| Rarely on any one day |  |

| **PP10: When I have to leave the baby/babies:** | Please tick |
| --- | --- |
| I usually feel rather sad (or it’s difficult to leave) |  |
| I often feel rather sad (or it’s difficult to leave) |  |
| I have mixed feelings of both sadness and relief |  |
| I often feel rather relieved (and it’s easy to leave) |  |
| I usually feel rather relieved (and it’s easy to leave) |  |

| **PP11: When I am with the baby/babies:** | Please tick |
| --- | --- |
| I always get a lot of enjoyment/satisfaction |  |
| I frequently get a lot of enjoyment/satisfaction |  |
| I occasionally get a lot of enjoyment/satisfaction |  |
| I very rarely get a lot of enjoyment/satisfaction |  |

| **PP12: When I am not with the baby/babies, I find myself thinking about the baby/babies:** | Please tick |
| --- | --- |
| Almost all the time |  |
| Very frequently |  |
| Frequently |  |
| Occasionally |  |
| Not at all |  |

| **MP13: When I am with the baby/babies:** | Please tick |
| --- | --- |
| I usually try to prolong the time I spend with him/her/them |  |
| Neither |  |
| I usually try to shorten the time I spend with him/her/them |  |

| **PP14: When I have been away from the baby/babies for a while and I am about to be with him/her/them again, I usually feel:** | Please tick |
| --- | --- |
| Intense pleasure at the idea |  |
| Moderate pleasure at the idea |  |
| Mild pleasure at the idea |  |
| No feelings at all about the idea |  |
| Negative feelings about the idea |  |

| **PP15: Over the past 3 months I have found myself just sitting looking at the sleeping baby/babies for periods of five minutes or more:** | Please tick |
| --- | --- |
| Very frequently |  |
| Frequently |  |
| A few times |  |
| Not at all |  |

| **PP16: I now think of the baby/babies as:** | Please tick |
| --- | --- |
| Very much my own baby/babies |  |
| A bit like my own baby/babies |  |
| Not really my own baby/babies |  |

| **PP17: Regarding the things that we have had to give up because of the baby/babies:** | Please tick |
| --- | --- |
| I find that I resent it quite a lot |  |
| I find that I resent it a moderate amount |  |
| I find that I resent it a bit |  |
| I don’t resent it at all |  |

| **PP18: Over the past 3 months, I have felt that I do not have enough time for myself or to pursue my own interests:** | Please tick |
| --- | --- |
| Almost all the time |  |
| Very frequently |  |
| Occasionally |  |
| Not at all |  |

| **PP19: Usually when I am with the baby/babies:** | Please tick |
| --- | --- |
| I am very impatient |  |
| I am a bit impatient |  |
| I am moderately patient |  |
| I am extremely patient |  |

**Edinburgh Postnatal Depression Scale**

**These questions assess your risk for depression. If you score highly, we will inform you and advise you to see your GP for further assessment and support if required.**

**Please tick one box only in answer to each question which comes closest to how you have felt over the last 7 days.**

| **EPDSP1: I have been able to laugh and see the funny side of things:** | Please tick |
| --- | --- |
| As much as I always could |  |
| Not quite so much now |  |
| Definitely not so much now |  |
| Not at all |  |

| **EPDSP2: I have looked forward with enjoyment to things:** | Please tick |
| --- | --- |
| As much as I ever did |  |
| Rather less than I used to |  |
| Definitely less than I used to |  |
| Hardly at all |  |

| **EPDSP3: I have blamed myself unnecessarily when things went wrong:** | Please tick |
| --- | --- |
| Yes, most of the time |  |
| Yes, some of the time |  |
| Not very often |  |
| No, never |  |

| **EPDSP4: I have been anxious or worried for no good reason:** | Please tick |
| --- | --- |
| No not at all |  |
| Hardly ever |  |
| Yes, sometimes |  |
| Yes, very often |  |

| **EPDSP5: I have felt scared or panicky for no very good reason:** | Please tick |
| --- | --- |
| Yes, quite a lot |  |
| Not quite so much now |  |
| Definitely not so much now |  |
| Not at all |  |

| **EPDSP6: Things have been getting on top of me:** | Please tick |
| --- | --- |
| Yes, most of the time I haven’t been able to cope at all |  |
| Yes, sometimes I haven’t been coping as well as usual |  |
| No, most of the time I have coped quite well |  |
| No, I have been coping as well as ever |  |

| **EPDSP7: I have been so unhappy that I have had difficulty sleeping:** | Please tick |
| --- | --- |
| Yes, most of the time |  |
| Yes, sometimes |  |
| Not very often |  |
| No, not at all |  |

| **EPDSP8: I have felt sad or miserable:** | Please tick |
| --- | --- |
| Yes, most of the time |  |
| Yes, sometimes |  |
| Not very often |  |
| No, not at all |  |

| **EPDSP9: I have been so unhappy that I have been crying:** | Please tick |
| --- | --- |
| Yes, most of the time |  |
| Yes, quite often |  |
| Only occasionally |  |
| No, never |  |

| **EPDSP10: The thought of harming myself has occurred to me:** | Please tick |
| --- | --- |
| Yes, quite often |  |
| Sometimes |  |
| Hardly ever |  |
| Never |  |

**Thank-you very much for taking the time to complete this questionnaire and being part of this study**

Detail of researchers

1. Dr Fiona Mackie,

Clinical Research Fellow,

Department of Maternal & Fetal Medicine, Floor 3,

Birmingham Women’s Hospital,

Edgbaston, Birmingham, B15 2TG.

Telephone No: 0121-626-4535

1. Professor M.D. Kilby,

Department of Maternal & Fetal Medicine, Floor 3,

Birmingham Women’s Hospital,

Edgbaston, Birmingham, B15 2TG.

Telephone No: 0121-627-2778

Patient Advice Liaison Service

Tel: 0121 627 2747

Email: [pals@bwnft.nhs.uk](mailto:pals@bwnft.nhs.uk)

This email address is being protected from spambots. You need JavaScript enabled to view it.

**Fetal Medicine Centre, Birmingham Women’s Foundation Trust,**

Metchley Park Road, Edgbaston, Birmingham. B15 2TG, Tel.No: 0121 627 2683
