# Supplementary material for: Parental attachment and depressive symptoms in pregnancies complicated by twin-twin transfusion syndrome: a cohort study
Source: BMC Pregnancy Childbirth. 2019 Dec 31;20:4. doi: 10.1186/s12884-019-2679-7 (PMC6938629; doi:10.1186/s12884-019-2679-7)
Supplement: Supplementary file 2 — Additional file 2. Additional methods [29, 41]. [file 12884_2019_2679_MOESM2_ESM.docx]

**Additional File 2** Additional methods information

In this work, parental attachment refers to either maternal or paternal attachment to the fetus(es) or infant(s) depending on whether the child is in-utero or has been born. As this was a sample of convenience, no power calculation was performed.

*Measures*

1. Parental Attachment Scale questionnaires

- Maternal Antenatal Attachment Scale (MAAS) 19 items, range of scores 19-95, sub-groups of ‘quality’ and ‘intensity’ of attachment.
- Paternal Antenatal Attachment Scale (PAAS) 16 items, range of scores 16-80, sub-groups of ‘quality’ and ‘intensity’ of attachment.
- Maternal Postnatal Attachment Scale (MPAS) 19 items, range of scores 19-95, sub-groups of ‘quality of attachment’, ‘absence of hostility’ and ‘pleasure in interaction’.
- Paternal Postnatal Attachment Scale (PPAS) 19 items, range of scores 19-95, sub-groups of ‘patience and tolerance’, ‘pleasure in interaction’ and ‘affection and pride’.

A higher score denotes greater attachment. These attachment tools have acceptable internal consistency, test-retest reliability, construct validity and have been demonstrated to be a valid measure of parento-fetal attachment and parento-infant attachment in numerous countries, including English-speaking countries as in this study (Condon 1998, Condon 2013). The majority of questions on the scale seemed appropriate, although 1 question in the MAAS and PAAS was difficult to interpret in the context of TTTS: “Since the diagnosis of TTTS when I think about the babies inside me I get feelings which are:” the responses vary from “Very sad” to “Very happy”. As the pregnancy is in danger at this point the question may not be able to discriminate between parents with high and low fetal attachment. The scoring of this question was not altered, in line with other studies which have used the MAAS in high-risk pregnancies (White 2008, Pisoni 2015). The reworded questionnaires were piloted on couples with twin pregnancies for sense prior to use in the actual study. As the maximum total score of the PAAS was different to the MAAS, MPAS and PPAS, the scores were converted into percentages of the maximum total score to allow comparison.

1. Edinburgh Depression Scale (EDS) and Edinburgh Postnatal Depression Scale (EPDS)

Depressive symptoms were assessed antenatally and postnatally using the EDS and EPDS respectively, which will be referred to as ‘EPDS’ irrespective of whether it was used antenatally or postnatally. The EDS and EPDS both consist of the same 10 questions and a 4-point self-rated scale of depressive symptoms. The total lowest score possible is 0, and maximum is 30 (Cox 1987). A higher score denotes greater depressive symptoms. A cut-off of 15 or more was used for maternal antenatal depression to give a sensitivity of 91% and specificity of 95% for indicating major depressive disorders in English speaking women, and 13 or more for maternal postnatal depression (Matthey 2006). Although the questionnaire is validated for use in fathers antenatally a cut-off for major depressive disorders has not been validated (Matthey 2006) so a cut-off of 12 or more was used for paternal antenatal depression as in Ramchandani et al. and Buist et al. (Buist 2003, Ramchandani 2008). A cut-off of 10 or more was used for paternal postnatal depression to give a sensitivity of 71.4% and specificity of 93.8% (Matthey 2001). This cut-off was 2 points lower than the validated antenatal cut-off, which is the difference between the maternal antenatal and postnatal cut-offs (Matthey 2006), but it is important to highlight that the paternal antenatal depressive symptoms cut-off may not necessarily be reflective of pathology. The EPDS has been validated for use in mothers and fathers antenatally and postnatally including English-speaking parents as in this study (Cox 1987, Murray 1990, Cox 1996, Matthey 2001, Matthey 2006).

*Missing data*

If a participant only completed the Attachment Scale, or EPDS portion of the questionnaire, their answers were included for the completed portion. Where one person in the couple did not complete the questionnaire, the answers of the other person were included in the analysis examining either mothers or fathers individually, but the couple was not included in the comparison between mothers and fathers. For single missing answers, median substitution was performed as indicated in **Error! Reference source not found.**, no other imputations were performed.

###

### Statistical analysis

All statistical analysis was performed in Stata (Stata, 2015 Release 13.1 StataCorp, Texas, USA). The Wilcoxon rank-sum test was performed to compare the maternal and paternal scores at each time point, and those with and without mental health problems. Fisher’s exact test was used to compare the proportion of mothers and fathers above the EPDS cut-off at each time point. To assess any difference longitudinally within the mothers or fathers who completed questionnaires at all 3 time points, the Shapiro-Wilk test of normality was performed, and the presence of outliers examined using box plots. All data were normally distributed except the paternal pre-FLA EPDS score, and the only outliers were 2 paternal attachment scores pre-FLA. Consequently one-way repeated-measures analysis of variance (ANOVA) was performed to assess maternal attachment and EPDS scores over time. If a significant difference was found, this was investigated by linear regression. Paternal attachment and EPDS scores were examined by the Kruskall-Wallis test.

Sub-group analysis was planned to assess the effect of those with past/current mental health problems, and those with one survivor compared to two survivors. A post-hoc sub-group analysis was performed to assess the effect of participants who did not complete the pre-FLA questionnaires before FLA and those who completed it immediately after FLA instead.
