# Supplementary material for: Parental attachment and depressive symptoms in pregnancies complicated by twin-twin transfusion syndrome: a cohort study
Source: BMC Pregnancy Childbirth. 2019 Dec 31;20:4. doi: 10.1186/s12884-019-2679-7 (PMC6938629; doi:10.1186/s12884-019-2679-7)
Supplement: Supplementary file 3 — Additional file 3. Additional results. [file 12884_2019_2679_MOESM3_ESM.docx]

**Additional File 3** Additional results

**Table 1 Questionnaires received at each time point and pregnancy outcome** w=weeks

| **Study ID** | **Pre-FLA** | **Post-FLA** | **Post-natal** | **Pregnancy outcome** | **Issues with questionnaire completion** |
| --- | --- | --- | --- | --- | --- |
| 2033 | y | y | y | 2 survivors born at 32w | None |
| 2036 | y | y |  | 1 survivor born at 35w, sIUFD at 20w | Father did not complete post-FLA |
| 2039 | y |  |  | 0 survivors, dIUFD at 23w | None |
| 2040 | y |  |  | 1 survivor born at 26w, 1 NND | None |
| 2041 | y |  |  | 1 survivor | None |
| 2042 | y | y |  | 2 survivors born at 30w | Maternal and paternal pre-FLA completed just after FLA |
| 2047 | y | y | y | 2 survivors born at 30w | None |
| 2054 | y |  |  | 2 survivors born at 32w | None |
| 2055 | y | y | y | 1 survivor born at 36w, sIUFD 17w | None |
| 2066 | y |  |  | 1 survivor born at 28w, sIUFD at 18w | Answer MAAS 1.3 missing (median substitution) |
| 2069 | y |  |  | 2 survivors born at 30w | None |
| 2071 | y | y | y | 2 survivors | None |
| 2078 | y |  |  | 0 survivors, dIUFD at 21w | None |
| 2095 | y |  |  | 1 survivor born at 31w | None |
| 2097 | y | y | y | 2 survivors | Maternal and paternal pre-FLA completed just after FLA |
| 2098 | y |  |  | 1 survivor born at 34w | None |
| 2101 | y |  |  | Unable to perform FLA | Maternal and paternal pre-FLA completed just after FLA. No maternal or paternal EPDS completed |
| 2102 | y |  |  | 0 survivors, sIUFD at 19w then TOP | None |
| 2105 | y |  |  | Unable to perform FLA | Answer MAAS 1.14 missing (median substitution) |
| **Study ID** | **Pre-FLA** | **Post-FLA** | **Post-natal** | **Pregnancy outcome** | **Issues with questionnaire completion** |
| 2107 | y |  |  | 0 survivors, dIUFD | None |
| 2116 | y |  |  | 0 survivors, PPROM at 18w then TOP | Father did not complete pre-FLA. Answer MAAS 1.16 missing (median substitution) |
| 2118 | y |  |  | 1 survivor born at 30w, sIUFD at 30w | Maternal and paternal pre-FLA completed just after FLA |
| 2119 | y |  |  | 2 survivors born at 31w | Maternal and paternal pre-FLA completed just after FLA |
| 2123 | y | y |  | 2 survivors born at 29w | Maternal and paternal pre-FLA completed just after FLA |
| 2125 | y |  |  | 0 survivors, dIUFD at 21w | Answer MAAS 1.15 missing (median substitution) |

### Mental health history of participants

At diagnosis of TTTS, one mother reported a current mental health problem (anxiety), and 6 mothers reported a combination of previous mental health problems (5 anxiety, 2 with concurrent depression, 1 postnatal depression, 1 post-traumatic stress disorder, 1 anorexia). Five fathers reported a current mental health problem at diagnosis of TTTS (1 depression, 4 anxiety, 2 with concurrent depression, 1 obsessive compulsive disorder), and 2 fathers reported a past mental health problem (1 anxiety, 1 depression). No new mental health problems were diagnosed during the study.

### Maternal and paternal attachment

**Table 2 Maternal and paternal attachment pre-fetoscopic laser ablation (FLA), post-FLA and postnatally**

NA: not applicable as sub-group does not exist in questionnaire. Median and IQR presented.

|  | **Maternal pre-FLA**  **(n=25)** | **Maternal post-FLA**  **(n=8)** | **Maternal postnatal**  **(n=5)** | **Paternal pre-FLA**  **(n=24)** | **Paternal post-FLA**  **(n=7)** | **Paternal postnatal**  **(n=5)** |
| --- | --- | --- | --- | --- | --- | --- |
| Total Attachment Scale score | 79  (74-82) | 82  (70.5-86.5) | 86.8 (74.4-88.2) | 63.5  (55-67) | 65  (58-68) | 82.1  (77.4-84.8) |
| Total Attachment Scale score as percentage of maximum possible | 83.2  (77.9-86.3) | 86.3 (74.2-91.1) | 91.4 (78.3-92.8) | 79.4 (68.8-83.8) | 81.3 (72.5-85) | 86.4 (81.5-89.3) |
| **Sub-groups of Attachment Scales** | | | | | | |
| Quality of attachment | 43  (40-45) | 44  (40-46.5) | 42.2  (42.2-42.2) | 35  (32-36.5) | 34  (32-37) | NA |
| Intensity of attachment | 30  (29-34) | 33  (26-34) | NA | 20  (15-21.5) | 21  (16-22) | NA |
| Pleasure in interaction | NA | NA | 24  (21-25) | NA | NA | 27.2 (26.9-27.3) |
| Absence of hostility | NA | NA | 19.6 (17.6-21) | NA | NA | NA |
| Patience and tolerance | NA | NA | NA | NA | NA | 36.2 (30.1-37.2) |
| Affection and pride | NA | NA | NA | NA | NA | 20  (19-20) |

|  Total attachment score (%)  Pre-FLA Post-FLA Postnatal  Maternal  Pre-FLA Post-FLA Postnatal  Paternal  **Figure 1 Box and whisker plots of maternal and paternal Attachment Scale scores as percentages of total maximum score pre-fetoscopic laser ablation (FLA), post-FLA and postnatally**  (n=25 mothers, n=25 fathers) No significant difference was found between mothers and fathers. Median and IQR presented. |
| --- |

**Table 3 Total parental attachment scores as a percentage of the maximum possible score pre-fetoscopic laser ablation (FLA), post-FLA and postnatally according to presence of mental health problems**

NA: not applicable as no participants in the sub-group. Median and IQR presented.

|  | **Maternal pre-FLA** | **Maternal post-FLA** | **Maternal postnatal** | **Paternal pre-FLA** | **Paternal post-FLA** | **Paternal postnatal** |
| --- | --- | --- | --- | --- | --- | --- |
| Current mental health problems | 85.26  n=1 | NA  n=0 | NA  n=0 | 81.25  (71.25-83.75)  n=5 | 87.50  n=1 | 96.00  n=1 |
| Past mental health problems | 78.95  (76.32-82.37)  n=6 | 71.58  (68.95-74.21)  n=2 | 78.32  n=1 | 85.00  (83.13-86.88)  n=2 | 83.75  n=1 | 89.26  n=1 |
| No history of mental health problems | 83.68  (79.21-87.11)  n=18 | 89.47 (85.26-91.32)  n=6 | 92.11  (87.68-94.00)  n=4 | 77.50  (67.50-82.50)  n=17 | 76.25  (72.50-81.25)  n=5 | 81.47  (79.42-83.95)  n=3 |

### Maternal and paternal depressive symptoms

| *  *  Total EPDS score  Pre-FLA Post-FLA Postnatal  Maternal  Pre-FLA Post-FLA Postnatal  Paternal  *  **Figure 2 Box and whisker plots of maternal and paternal Edinburgh Postnatal Depression Scale (EPDS) scores pre-fetoscopic laser ablation (FLA), post-FLA and postnatally**  (n=24 mothers, n=24 fathers) *p<0.05 pre-FLA, and post-FLA, when mothers were compared to fathers. Median and IQR presented. |
| --- |

**Table 4 Total maternal and paternal Edinburgh Postnatal Depression Scale (EPDS) scores pre-fetoscopic laser ablation (FLA), post-FLA and postnatally according to presence of mental health problems**

|  | **Maternal pre-FLA** | **Maternal post-FLA** | **Maternal postnatal** | **Paternal pre-FLA** | **Paternal post-FLA** | **Paternal postnatal** |
| --- | --- | --- | --- | --- | --- | --- |
| Current mental health problems | NA | NA | NA | 12^†^  (10-15) | 9 | 2 |
| Above cut-off n/N (%) | 0/0 | 0/0 | 0/0 | 3/5  (60) | 0/1  (0) | 0/1  (0) |
| Past mental health problems | 17  (11-20) | 23.5* (20.75-26.25) | 3 | 6 | 3 | 1 |
| Above cut-off n/N (%) | 4/6  (66.6) | 2/2  (100) | 0/1  (0) | 0/1  (0) | 0/1  (0) | 0/1  (0) |
| No history of mental health problems | 10  (7-16.75) | 10*  (9.25-13.75) | 5.5  (3.5-7.5) | 7^†^  (4-9) | 6  (6-10) | 9  (6-10.5) |
| Above cut-off n/N (%) | 6/18 (33.33) | 2/6 (33.33) | 0/4  (0) | 3/17 (17.65) | 1/5  (20) | 1/3  (33.33) |

*p<0.05 maternal post-FLA: past mental health problems vs no mental health problems †p<0.05 paternal pre-FLA: current mental health problems vs no mental health problems. NA: not applicable as no participants in the sub-group. Median (IQR) presented.
